# Effect of forest planting patterns on the formation of soil organic carbon during litter lignocellulose degradation from a microbial perspective

**DOI:** 10.3389/fmicb.2023.1327481

**Published:** 2023-12-22

**Authors:** Di Wu, Changwei Yin, Yuxin Fan, Haiyu Chi, Zhili Liu, Guangze Jin

**Affiliations:** ^1^Center for Ecological Research, Northeast Forestry University, Harbin, China; ^2^Key Laboratory of Sustainable Forest Ecosystem Management, Ministry of Education, Northeast Forestry University, Harbin, China; ^3^Northeast Asia Biodiversity Research Center, Northeast Forestry University, Harbin, China

**Keywords:** metagenomics, metabolomics, litter decomposition, soil organic carbon formation, urban forest

## Abstract

Litter decomposition is an important source of soil organic carbon, and it plays a key role in maintaining the stability of forest ecosystems. The microbial mechanism of soil organic carbon (SOC) formation in different urban forest planting patterns during litter lignocellulose degradation is still unclear. The key genes, microbes, and metabolites in the process of lignocellulose degradation and SOC formation were determined by metagenomics and metabolomics in different litter decomposition layers and soil layers in different urban forest planting patterns, including three types of broadleaf forests (BP forests), three types of coniferous forests (CP forests), and two types of mixed coniferous and broadleaf forests (MCBP forests). The results indicated that the cellulose, hemicellulose, and lignin concentrations from the undecomposed layer to the totally decomposed layer decreased by 70.07, 86.83, and 73.04% for CP litter; 74.30, 93.80, and 77.55% for BP litter; and 62.51, 48.58, and 90.61% for MCBP litter, respectively. The soil organic carbon of the BP forests and MCBP forests was higher than that of the CP forests by 38.06 and 94.43% for the 0–10 cm soil layer and by 38.55 and 20.87% for the 10–20 cm soil layer, respectively. Additionally, the gene abundances of glycoside hydrolases (GHs) and polysaccharide lyases (PLs) in the BP forests were higher than those in the MCBP forests and CP forests. Amino acid metabolism, sugar metabolism, TCA metabolism, and cAMP signaling metabolism were mainly between the CP forests and BP forests, while the TCA cycle, pyruvate metabolism, phenylalanine metabolism, and tyrosine metabolism were mainly between the BP forests and MCBP forests during litter decomposition. Additionally, ammonia nitrogen and hemicellulose were key factors driving SOC formation in the CP forests, while ammonia nitrogen, hemicellulose, and lignocellulose-degrading genes were key factors driving SOC formation in the BP forests. For the MCBP forests, cellulose, pH, ammonia nitrogen, and lignin were key factors driving SOC formation. Our findings revealed that the BP forests and MCBP forests had stronger lignocellulose degradation performance in the formation of SOC. This study provided a theoretical basis for the flow and transformation of nutrients in different urban forest management patterns.

## 1 Introduction

The forest ecosystem is one of the main components of terrestrial ecosystems and plays a significant role in the global carbon (C) cycle (Almeida et al., 2021). Soil organic carbon (SOC), as an important source of C, affects plant productivity and forest ecosystem diversity and stability (Jones et al., 2005). In forest ecosystems, plants provide primary C input into the soil, where leaf litter is decomposed to form humus and SOC by the action of microbes or abiotic polymerization (Zhou et al., 2015; Shi et al., 2018). Urban forests can provide many ecosystem services, such as improving air quality, sustaining biodiversity, and increasing C fixation (Roeland et al., 2019; Xu et al., 2021). In addition, urban forests also affect organic carbon components and then maintain ecosystem stability and mitigate climate change, thereby improving urban living quality and environments (Wang et al., 2023). Different urban forest planting patterns have different dominant plant species and compositions, resulting in variations in the substrate composition and quality of litter, soil properties, and microbial communities (Xu et al., 2021). Mixed-species forests with multiple species increase tree species diversity and improve the likelihood of finding species with complementary functional features for maximizing resource usage, according to the niche complementary hypothesis (Cassart et al., 2017). Meanwhile, mixed-species forests can increase forest productivity and improve soil microenvironments (Ding et al., 2023). In addition, plant tree species diversity (mixed planting of broadleaved tree species and coniferous tree species) alters the quality of litter (different C:N and lignin:N ratios) and then affects downstream litter decomposition metabolites on SOC formation. In these litter decomposition processes, the lignocellulosic degradation ratio was more rapid in broadleaf tree species litter than in coniferous tree species litter (Lyu et al., 2023). Another explanation is that variations in soil properties and microbial communities in mixed-species forests can strongly influence SOC formation (Cassart et al., 2017). Additionally, mixed-species forests influence litter substrate properties, sugar metabolism, and amino acid metabolism and then influence the SOC formation pathway, thereby influencing SOC formation and soil fertility (Wang et al., 2019; Wang W. et al., 2020; Ding et al., 2023).

Previous studies have clearly shown the process by which plant litter-derived C is transferred into SOC via microbial transformation, emphasizing the key role of microbes (Liang et al., 2017, 2019). Microbes can release extracellular enzymes to degrade litter's lignocellulosic components. The produced metabolites are closely linked to shifts in soil C storage (Veres et al., 2015). Microbial traits and functional groups vary with the chemical nature and substrate composition of litter and soil properties, thus influencing SOC formation (Wang Y. et al., 2020). Plant leaf litter is rich in lignocellulose, including cellulose, hemicellulose, lignin, and chitin. Thus, lignocellulosic degradation is a very important process for the nutrient transformation of litter (Wang Y. et al., 2020). In these processes, polysaccharides derived from cellulose and hemicellulose-degrading products are hydrolyzed by a complex set of glycoside hydrolases and auxiliary enzymes (Valášková et al., 2007; Wu et al., 2023). Additionally, lignin is degraded by oxidative enzymes and hydrolytic enzymes and then contributes to the formation of humus and SOC (Jiang et al., 2021; Cao et al., 2022). Lignocellulose-degrading enzymes are encoded by a variety of functional genes (Detain et al., 2022; Wu et al., 2023). The production of lignocellulose-degrading enzyme-associated functional genes is greatly influenced by the substrate composition of litter and microbial properties (Xu et al., 2022). The higher abundance of functional genes increases the production of lignocellulose-degrading enzymes, which determine the lignocellulosic degradation performance and then influence lignocellulose-degrading products, thus influencing humus and SOC formation (Wu et al., 2023). Appropriate soil factors and environmental conditions can promote the production of lignocellulose-degrading genes, which is conducive to the production of lignocellulases (Wu et al., 2023). Thus, how different types of planting forest litter influence lignocellulose-degrading genes and microbial community composition in the lignocellulose degradation pathway during litter decomposition is largely unknown.

Lignocellulose-degrading products are composed of multiple monomers, oligomers, and multimers, such as monosaccharides, amino acids, phenols, aldehydes, ketones, benzene, and organic acids (Voeller et al., 2017; Thomas et al., 2020). Among them, cellulose-degrading products and hemicellulose-degrading products are composed of different types of monosaccharides and polysaccharides, such as glucose, xylose, mannose, arabinose, and microbes derived from sugars, which can provide adequate nutrients for microbes (Hu et al., 2019). These sugars are not only key nutrient sources but also enter the cell inside to form aromatic amino acids and polyphenols in the shikimic acid cycle, which is a key precursor for the formation of humus and SOC (Wu et al., 2023). Moreover, polyphenols, quinones, benzenes, and aromatic compounds are derived from lignin degradation and are considered vital raw materials and key precursors for the synthesis of humus and SOC (Irene et al., 2014). It is anticipated that the initial chemical discrepancies in the external source of C of different types of litter will converge through microbial anabolism, according to the chemical convergence hypothesis (Liang et al., 2017). This emphasizes the entire metabolic process of microorganisms, including gene expression, extracellular enzyme secretion, and metabolite production from gene-protein-metabolism levels (Nemergut et al., 2010; Irene et al., 2014). However, there are significant knowledge gaps about the key pathways and the relationship between genes and metabolism in litter lignocellulose degradation and SOC formation. The factors influencing the formation of SOC in different forest planting patterns during litter decomposition are largely unknown.

Thus, in this study, we explored the processes of lignocellulose degradation in litter layers and SOC formation in soil layers in eight typical forests of three plantation types, including coniferous forests, broadleaved forests, and mixed forests. We aimed to test the following hypotheses: (1) significant differences in microbial communities and lignocellulose degradation in litter layers and soil layers in different forest planting patterns may be present, (2) differential microbial functional genes and metabolites involved in lignocellulose degradation result in the difference in SOC formation in different forest planting patterns, and (3) key factors affecting the formation of SOC in different forests were significantly different. To explore these hypotheses, metagenomics and metabolomics were applied to investigate the process and pathway of litter decomposition and SOC formation in forest planting patterns. Therefore, the application of this research provides a theoretical basis for better management practices for forest planting patterns.

## 2 Materials and methods

### 2.1 Sample collection

In this study, samples were collected at the Harbin Urban Forestry Demonstration Base of Northeast Forestry University (44°04′-46°40′ N, 125°42′-130°10′ E). The original vegetation of the forest sample site was herbaceous. The soil type was black soil. In the 1950s, different types of artificial forests were built. All planting forest areas were 4,900 m^2^. The samples in all forests were collected from 8 to 20 October 2022. The city has a temperate continental monsoon climate. In this area, the annual average temperature is 3.5°C, the annual average rainfall is 534 mm, and the annual average humidity is 67%. All forest sample plots were set randomly as 20 m × 20 m quadrats. Litter and soil samples were collected from the center of each small plot (5 m × 5 m). Five small plots (5 m × 5 m) in each quadrat were set up. In this plot, five types of samples were collected, including three litter layers and two soil layers. Five different types of samples were randomly selected, and the triplicate samples were mixed to represent the litter samples and soil samples. The collected samples were placed on ice and brought back to the laboratory. To remove stones, roots, and plant debris, each soil sample was passed through a 2-mm sieve. Eight forest plantations were chosen in this study, including three coniferous forests, three broadleaved forests, and two mixed forests. The three coniferous forests consisted of *Pinus tabulaeformis* var. *mukdensis* (HPYS forest), *Larix gmelinii* (LYS forest), and *Pinus sylvestris* var. *mongolica* (ZZS forest). The three broadleaved forests consisted of *Juglans mandshurica* (HTQ forest)*, Fraxinus mandshurica* (SQL forest), and *Quercus mongolica* (MGL forest). Of the two mixed forests, one consisted of *Picea koraiensis, Juglans mandshurica, Larix gmelinii*, and *Fraxinus mandschurica* (ZKHJ forest), and the other consisted of *Betula platyphylla, Pinus koraiensis, Phellodendron amurense*, and *Fraxinus mandschurica* (BH forest). The physical and chemical properties of the forest litter and soil samples are shown in Table 1.

**Table 1:** Basic physical and chemical properties of different forest samples.

**Groups**	**pH**	**SOC (mg/g)**	**${\text{NH}}_{4}^{+}$-N (mg/g)**	**${\text{NO}}_{3}^{-}$N (μg/g)**	**TC (mg/kg)**
CP. A	4.88 ± 0.21	471.29 ± 27.34	0.86 ± 0.02	5.24 ± 0.007	477.32 ± 16.47
CP. B	5.95 ± 0.32	384.18 ± 25.65	0.22 ± 0.03	2.51 ± 0.0008	326.50 ± 17.34
CP. W	5.42 ± 0.23	253.06 ± 20.60	0.039 ± 0.002	1.73 ± 0.0005	204.65 ± 13.34
CP. S	5.32 ± 0.26	98.07 ± 14.50	0.10 ± 0.001	1.40 ± 0.0001	44.73 ± 4.44
CP. D	5.71 ± 0.19	74.07 ± 18.09	0.13 ± 0.02	1.16 ± 0.0003	18.09 ± 9.05
BP. A	5.43 ± 0.26	348.17 ± 15.24	1.85 ± 0.23	14.89 ± 0.007	403.32 ± 10.34
BP. B	5.77 ± 0.22	268.42 ± 19.14	0.85 ± 0.002	4.14 ± 0.0012	249.33 ± 18.34
BP. W	6.92 ± 0.28	102.07 ± 10.78	0.17 ± 0.006	1.97 ± 0.0014	67.57 ± 15.56
BP. S	6.78 ± 0.26	87.24 ± 12.09	0.09 ± 0.001	1.25 ± 0.0001	42.09 ± 8.93
BP. D	6.38 ± 0.28	88.27 ± 10.67	0.09 ± 0.001	1.19 ± 0.0001	20.09 ± 5.76
MCBP. A	6.10 ± 0.25	303.71 ± 10.67	1.80 ± 0.06	14.86 ± 0.015	292.28 ± 20.63
MCBP. B	7.45 ± 0.04	193.19 ± 12.98	0.70 ± 0.008	3.43 ± 0.0025	126.94 ± 23.76
MCBP. W	7.17 ± 0.02	156.22 ± 10.76	0.23 ± 0.002	1.13 ± 0.0005	136.36 ± 7.35
MCBP. S	6.90 ± 0.27	99.11 ± 15.76	0.10 ± 0.002	1.75 ± 0.0005	50.64 ± 2.09
MCBP. D	6.51 ± 0.02	68.33 ± 3.01	0.095 ± 0.003	1.21 ± 0.0002	12.28 ± 6.77

SOC, soil organic carbon; TC, total carbon.

A total of 40 different samples were selected from two types of samples, including three litter layers and two soil layers. The leaf litter layers were composed of a relatively undecomposed layer (A), a semi-decomposed layer (B), and a totally decomposed layer (W). The soil layers were composed of soil from 0 to 10 cm (S) and soil from 10 to 20 cm (D), which was topsoil. Different types of litter decomposition levels were identified according to the study by Sato et al. (2004), which represented the state of decomposition of litter. The undecomposed layer of litter indicated that the color and appearance of the litter had not changed (Sato et al., 2004). The semi-decomposed layer of litter indicated that the litter was degraded by microbes, and the shape and color could be altered, but the original tissue could be discerned (Sato et al., 2004). The totally decomposed layer of litter indicated that the color was dark brown and the shape was indistinguishable, which was equivalent to the humus and soil mixture (Sato et al., 2004). In the coniferous forests, for the undecomposed layer, semi-decomposed layer, and totally decomposed layer of litter, the 0–10 cm and 10–20 cm soil layers were named CP. A, CP. B, CP. W, CP. S, and CP. D. In the broadleaved forests, for the undecomposed layer, semi-decomposed layer, and totally decomposed layer of litter, the 0–10 cm and 10–20 cm soil layers were named BP. A, BP. B, BP. W, BP. S and BP. D. In the mixed forests, for the undecomposed layer, semi-decomposed layer, and totally decomposed layer of litter, the 0–10 cm and 10–20 cm soil layers were named MCBP. A, MCBP. B, MCBP. W, MCBP. S, and MCBP. D. A total of 300 g of each sample was collected; one sample was air-dried for basic physicochemical indicator determination, and the other sample was kept at −80°C in a refrigerator for microbial analysis. Table 2 shows the basic characteristics in all forests.

**Table 2:** Basic characteristics of different types of forests.

**Forest types**	**Stand age (A)**	**Average height (m)**	**Average DBH (cm)**	**Density (individual/ha)**	**Basel area (m^2^/ha)**
CP forest	63	16.07	20.02	525	26.84
BP forest	66	15.24	22.86	500	36.89
MCBP forest	62	10.08	17.56	575	22.56

### 2.2 DNA extraction and metagenomic analysis

The DNA of litter and soil samples in all forests was extracted using a DNA kit (Omega, Shanghai, China). The quality and concentration of DNA samples were examined by a Qubit® 2.0 Flurometer, and the samples were submitted to Novogene (Beijing, China) for shotgun metagenomics sequencing based on Illumina NovaSeq (Illumina Inc., San Diego, CA, USA) to perform paired-end sequencing.

The open reading frame (ORF) prediction of contigs within the resulting contigs was carried out by utilizing MetaGene (http://metagene.cb.k.u-tokyo.ac.jp/). The sequences whose nucleic acid lengths were larger than or equivalent to 100 bp were screened and translated into amino acid sequences utilizing the National Center for Biotechnology Information (NCBI) translation tables. The predicted genes with 90% sequence identity were grouped on CD-HIT (http://www.bioinformatics.org/cd-hit/). The quality control was analyzed using the representative sequences with 95% identity utilizing SOAPaligner (http://soap.genomics.org.cn/) (Li et al., 2022).

To study the variation and diversity of lignocellulose-degrading enzymes encoding genes and SOC formation-related genes, we analyzed the high-quality unassembled reads against the Carbohydrate Active Enzyme (CAZy) database and the Kyoto Encyclopedia of Genes and Genomes database (KEGG) (Wang Y. et al., 2020).

### 2.3 Metabolomics sequencing

Metabolites in all forest litter and soil samples were measured by gas chromatograph mass spectrometry (GC-MS). A total of 600 mg of sample was ground, sonicated, centrifuged, dried, and derivatized (Dunn et al., 2011). After drying, the sample was added to a pyridine solution of methoxamine hydrochloride for the oxime reaction. Then, 50 μl of BSTFA derivatization reagent and 20 μl of hexane were added to perform GC-MS metabolomics analysis (Dunn et al., 2011).

The GC-MS procedure was a DB-5MS capillary column, and the carrier gas was high-purity helium. The flow rate was 1 ml/min, and the inlet temperature was 260°C. The injection volume was 1 μl, with no split injection and a solvent delay of 4.8 min. An initial temperature of 60°C was programmed to be raised at 8°C/min until 125°C was reached, followed by 210°C at 8°C/min and 305°C at 20°C/min for 5 min. The obtained raw metabolite data were addressed with MS-DIAL based on the LUG database (Dunn et al., 2011; Wu et al., 2023).

### 2.4 Determination of soil basic properties

The pH value was determined using a digital pH meter (Zhang et al., 2021). By oxidizing the potassium dichromate method, the SOC concentration was determined (Zhang et al., 2021). The concentrations of cellulose, hemicellulose, and lignin were determined according to the kit method (Martyna et al., 2017). The lignin concentration was acetylated and then determined at 280 nm (Martyna et al., 2017). The cellulose concentration was decomposed under acidic conditions to generate β-glucose and then β-furfural compounds, which were determined at 620 nm (Martyna et al., 2017). In addition, the hemicellulose concentration was converted into reducing sugars under acid treatment conditions and was determined at 540 nm (Martyna et al., 2017). The fresh sample was utilized for identifying nitrate nitrogen (${\text{NO}}_{3}^{-}$-N) concentration by the colorimetric method (Nie et al., 2018). The ammonium nitrogen (${\text{NH}}_{4}^{+}$-N) concentration was evaluated by colorimetry using Nessler's reagent (Nie et al., 2018).

### 2.5 Statistical analysis

Statistical analysis of lignocellulosic components and other related indicators was performed in SPSS 22.0, which was set to *P* < 0.05. The visualization of data was performed by the Origin 2023 software. The nine-quadrant plot was used to identify key lignocellulose-degrading enzyme-encoding genes and metabolites, which was carried out on the cloud platform (Xie et al., 2023). The regions of interest in the nine-quadrant plot were in the first, third, seventh, and ninth quadrants (levels of genes or metabolites with 2-fold differences in different forests, |Log2FC| ≥ 1) (Xie et al., 2023). Correlation analysis and random forest modeling were performed using the “Hmisc,” “Corrplot,” and “RandomForest” packages in R Studio (Wu et al., 2023). The differential significance analysis of lignocellulose-degrading metabolites and genes was carried out in a cloud platform (Wu et al., 2023). An ANOVA was used to assess the experimental data. Differences among samples were analyzed using the LSD all-pairwise comparisons test. A structural equation model (SEM) was performed using the Amos 23 software (Wu et al., 2022).

## 3 Results

### 3.1 Microbial taxa

**Figure 1:**
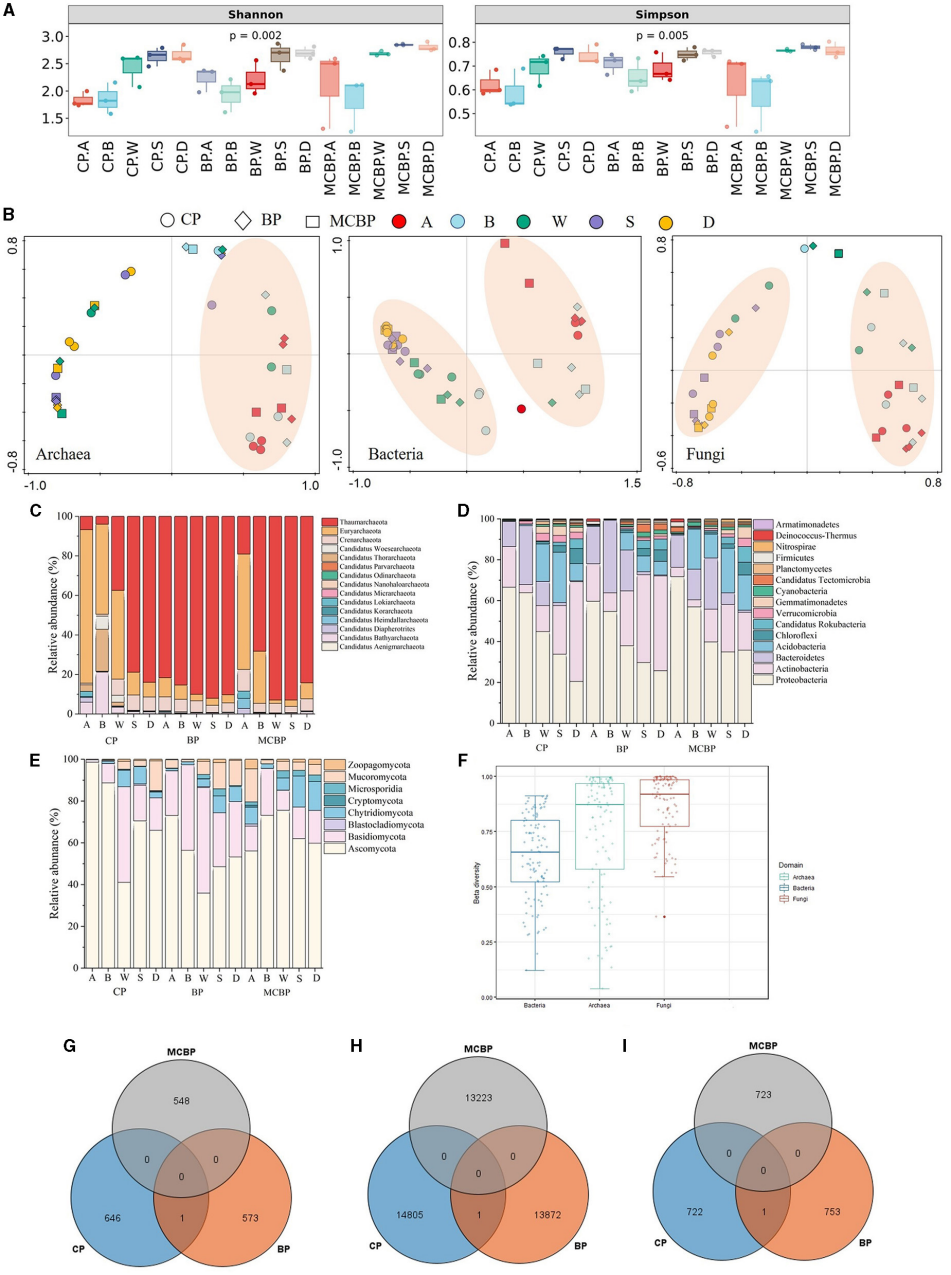
Variations in microbial taxa in different forests. **(A)** Microbial diversity. **(B)** PCoA analysis of microbiomes. **(C)** The relative abundance of archaeal communities at the phylum level. **(D)** The relative abundance of bacterial communities at the phylum level. **(E)** The relative abundance of fungal communities at the phylum level. **(F)** β-diversity of microbiomes in all forests. **(G)** Archaeal shared and special species in all forests. **(H)** Bacterial shared and special species in all forests. **(I)** Fungal shared and special species in all forests.

As shown in Figure 1A, the microbial diversity of the BP forests and MCBP forests was higher than that of the CP forests. The microbial diversity in the BP forests was not significantly different from that in the MCBP forests (*P* > 0.05) (Figure 1A). The results of principal coordinate analysis (PCoA) showed that the microbiome community structure, including that of archaea, bacteria, and fungi, of all forests was similar at the same soil layer depth. The results revealed that the microbiomes of the MCBP forest and BP forest samples were clustered together, while the microbiomes of the CP forest samples were far from them (Figure 1B). In addition, β-diversity in all forests was ranked in order of archaea, fungi, and bacteria (Figure 1F). Additionally, bacterial communities at the species level exhibited significant differences among different forests (ANOSIM R = 0.07, *P* < 0.05, Supplementary Figure S1b), while there was no significant difference in archaeal communities (ANOSIM R = 0.026, *P* > 0.05, Supplementary Figure S1a) or fungal communities (ANOSIM R = 0.016, *P* > 0.05, Supplementary Figure S1c).

As shown in Figure 1D, the bacterial phyla in all forests were dominated by Proteobacteria, Actinobacteria, Bacteroidetes, Acidobacteria, Chloroflexi, and Candidatus Rokubacteria. The relative abundance of Proteobacteria in the MCBP forest litter layer was 24.35% and 13.23% higher than that of the BP forests and CP forests, respectively (Figure 1D). The relative abundance of Actinobacteria in the CP forest soil layer was 61.01% and 7.96% higher than that of the MCBP forests and BP forests, respectively (Figure 1D). Actinobacteria were the dominant bacteria in the CP forest soil layer, and Bacteroidetes were the dominant bacteria in the BP forest litter layer (Figure 1D). Acidobacteria and Candidatus Rokubacteria were the dominant bacteria in the MCBP forests (Figure 1D). In addition, Candidatus Tectomicrobia was the key bacteria in the BP forests (Figure 1D). In addition, the most dominant fungal phyla in all forests were Ascomycota, Basidiomycota, Chytridiomycota, Mucoromycota, and Zoopagomycota (Figure 1E). The relative abundance of dominant species varied significantly in different forests (*P* < 0.05). The relative abundance of Ascomycota in the CP forests and MCBP forests was 83.12% and 38.75% higher than that of the BP forests, respectively. The relative abundance of Basidiomycota in the BP forests was higher than that in the CP forests and MCBP forests. Additionally, the relative abundance of Chytridiomycota in the BP forests and MCBP forests in the totally decomposed layer and the soil layers was higher than that in the CP forests. Furthermore, the most dominant archaeal phyla in all forests were Thaumarchaeota, Euryarchaeota, Crenarchaeota, and Candidatus Woesearchaeota (Figure 1C). The relative abundance of Thaumarchaeota in the BP forests was higher than that in the CP forests and MCBP forests. However, the relative abundance of Euryarchaeota in the CP forest and MCBP forest litter layers was higher than that in the BP forests (Figure 1C).

The Venn diagram revealed that the specific archaeal and bacterial species numbers in the CP forest were higher than those in the BP forests and MCBP forests (Figures 1G–I). However, the specific fungal species number in the BP forests was higher than that in the CP forests and MCBP forests. These results demonstrated that there were significant differences in microbial communities among the different types of forests (*P* < 0.05).

### 3.2 Variation in nutrient substrates

#### 3.2.1 Lignocellulose degradation during litter decomposition

**Figure 2:**
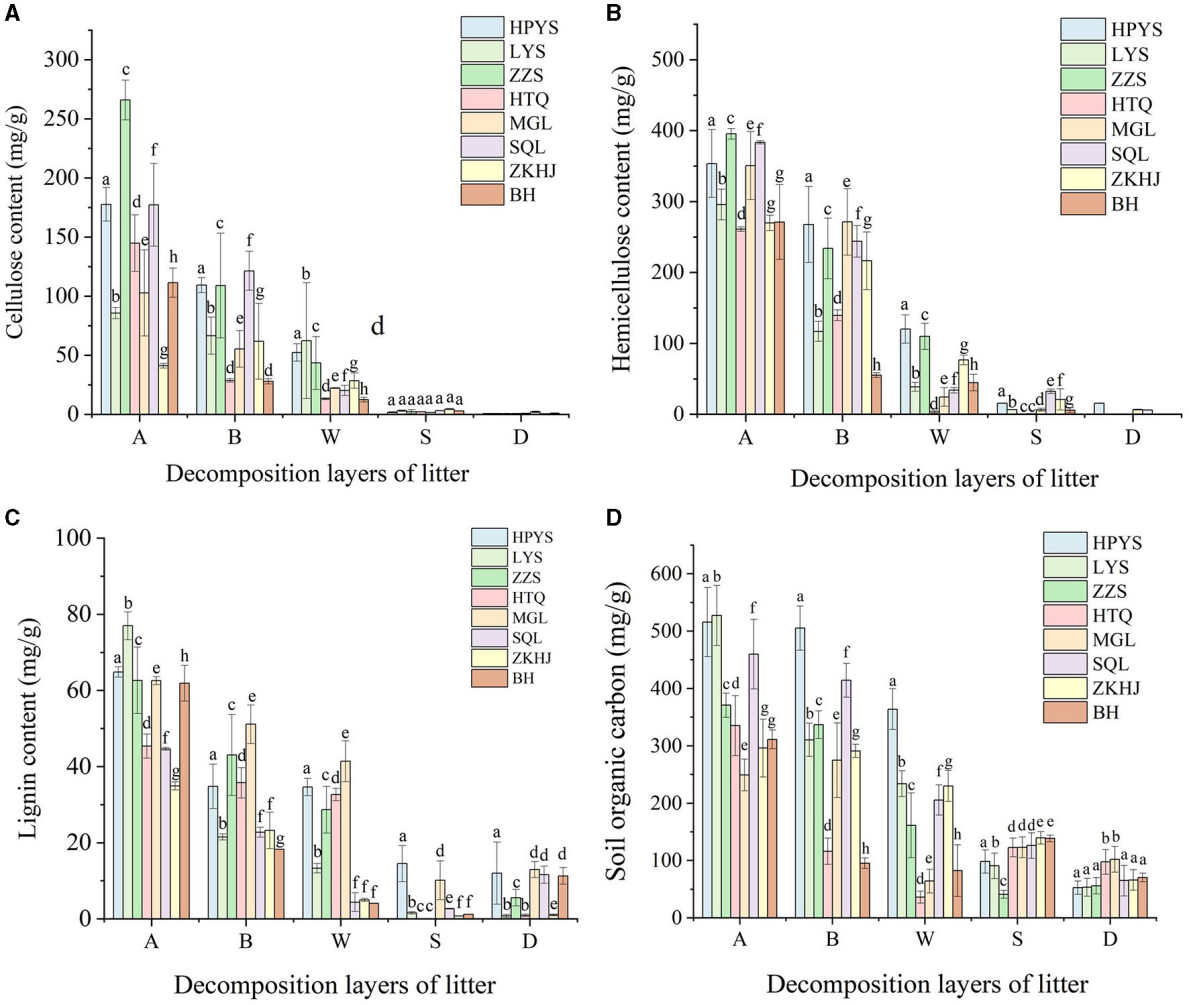
Changes in nutrient substrates in different forests. **(A)** Cellulose concentration. **(B)** Hemicellulose concentration. **(C)** Lignin concentration. **(D)** SOC concentration.

Cellulose and hemicellulose concentrations decreased in all forests with the depth of the soil layers (Figures 2A, B). The cellulose concentration in HPYS forest, LYS forest, ZZS forest, HTQ forest, MGL forest, SQL forest, ZKHJ forest, and BH forest from the undecomposed layer to the totally decomposed layer in litter decreased by 70.48, 27.18, 83.62, 90.82, 78.29, 88.53, 28.17, and 88.72%, respectively (Figure 2A). In addition, the hemicellulose concentration in HPYS forest, LYS forest, ZZS forest, HTQ forest, MGL forest, SQL forest, ZKHJ forest, and BH forest from the undecomposed layer to the totally decomposed layer in litter decreased by 66.02, 86.95, 72.24, 98.66, 93.07, 91.16, 71.57, and 83.51%, respectively (Figure 2B). In general, the BP forests showed the lowest cellulose and hemicellulose concentrations. Compared with the CP forests, the higher decomposition performance of cellulose and hemicellulose in the MCBP forests was significant (*P* < 0.05). In addition, the lignin concentration in the MCBP forests was lowest in the totally decomposed litter layer (Figure 2C). Only the lignin concentration in the soil layer exhibited an upward trend in all forests. The lignin concentration of the BP forests in the soil layer was higher than that of the MCBP forests and CP forests (Figure 2C).

#### 3.2.2 Variation in soil organic carbon

The SOC and total carbon (TC) concentrations in all forests showed a decreasing trend in litter layers (Table 1, Figure 2D). The SOC concentration from the undecomposed layer to the totally decomposed layer in litter decreased by 29.42, 55.65, 56.56, 78.29, 74.22, 55.30, 22.35, and 73.50% for HPYS forest, LYS forest, ZZS forest, HTQ forest, MGL forest, SQL forest, ZKHJ forest, and BH forest, respectively (Figure 2D). The SOC was derived from litter-C, leading to a decrease in SOC. In general, the BP forests had the highest SOC and TC decomposition performance in the litter layers (Table 1). For the soil layer from 0 to 10 cm, the SOC and TC concentrations of the MCBP forests were higher than those of the BP forests and CP forests (Figure 2D). Moreover, the SOC concentration of the 10–20 cm soil layer of the BP forests was the highest.

### 3.3 Metagenomics sequencing in the decomposition process of litter

#### 3.3.1 Functional annotation of carbohydrate -active enzymes

**Figure 3:**
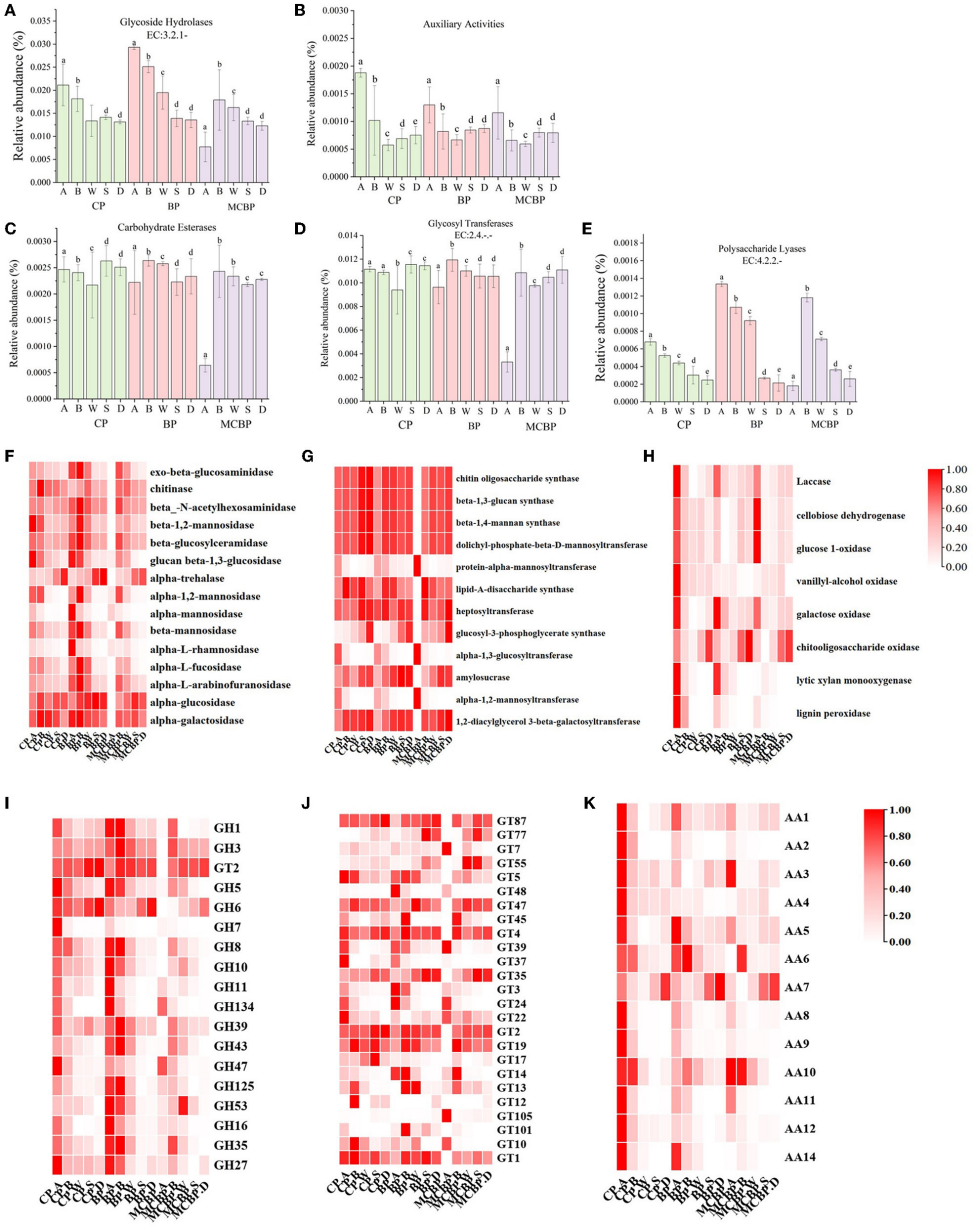
Macrogenomic analysis of changes in genes encoding lignocellulolytic enzymes. **(A)** Glycoside hydrolases. **(B)** Auxiliary activities. **(C)** Carbohydrate esterases. **(D)** Glycosyl transferases. **(E)** Polysaccharide lyases. **(F)** GHs family enzymes encoding genes. **(G)** GTs family enzymes encoding genes. **(H)** AAs family enzymes encoding genes. **(I)** GHs genes. **(J)** GTs genes. **(K)** AAs genes.

Lignocellulose-degrading enzymes are mainly affiliated with carbohydrate-active enzymes (CAZy), which are classified according to the CAZy database into five classes: glycoside hydrolases (GHs), auxiliary activities (AAs), carbohydrate esterases (CEs), glycosyl transferases (GTs), and polysaccharide lyases (PLs) (Wu et al., 2023). The gene abundances of GHs (organic C decomposition) and PLs (polysaccharide decomposition) in the BP forests were higher than those in the MCBP forests and CP forests (Figures 3A, E). In addition, the gene abundance of AAs (polysaccharide cleavage auxiliary enzymes and lignin-degrading enzymes) in the CP forests was higher than that in the BP forests and MCBP forests (Figure 3B). However, the gene abundances of CEs (hydrolysis of carbohydrate esters) and GTs (organic C biosynthesis) were not significantly different in all forests (*P* > 0.05) (Figures 3C, D).

#### 3.3.2 Microbial enzymes involved in lignocellulose degradation and C biosynthesis

The encoding genes of the GHs family (EC: 3.2.1.-), GTs family (EC: 2.4.-) and AAs family-related enzymes were analyzed (Figures 3F–H). In general, the gene abundance of the GHs family (*GH1, GH2, GH6, GH39*, and *GH53*) in the BP forests and MCBP forests was higher than that in the CP forests in all forests (Figure 3F). The gene abundance of the AAs family in the CP forests was higher than that in the BP forests and MCBP forests (Figures 3H, K). In addition, the gene abundance of GTs family (*GT4, GT39, GT22*, and *GT14*) in the CP forests and MCBP forests was higher than in the BP forests. The gene abundance of the GTs family (*GT87, GT47, GT4, GT35, GT2*, and *GT19*) in the totally decomposed litter layer and soil layer in BP forests and MCBP forests was the highest (Figure 3G). Furthermore, the gene abundance for the AAs family (*AA1, AA5, AA6, AA7*, and *AA10*) in all forest litter undecomposed layers and soil layers was the highest (Figure 3K).

### 3.4 Metabolome analysis of lignocellulose degradation

#### 3.4.1 Excavation of differential metabolites

The results showed that there were a total of 217 and 524 metabolites in the litter and soil samples, respectively, based on metabolomics sequencing. Metabolites for the soil layers and litter layers included 11, 23 phenols; 12, 33 benzene and substituted derivatives; 28, 65 carboxylic acids; 3, 0 phenolic acids; 33, 73 long-chain carbon organic acids; 9, 26 organic acids; 57, 109 sugars; 3, 0 ketones; and 8, 9 amino acids. PCoA based on Bray-Curtis distances illustrated that the metabolites from litter layers and soil layers were not significantly different (*P* > 0.05, PERMANOVA by Adonis) (Supplementary Figure S3).

**Figure 4:**
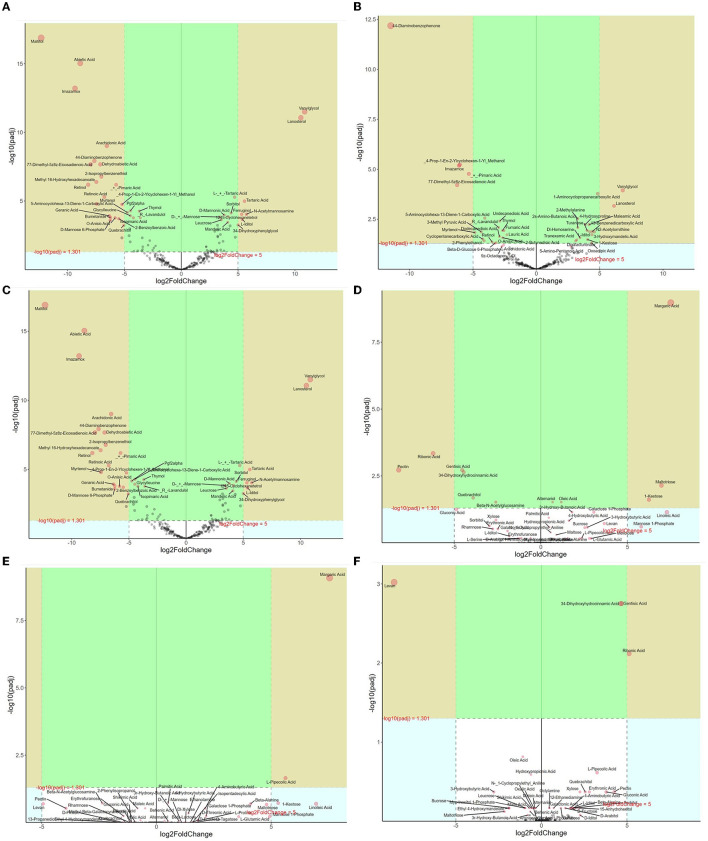
Volcano map analysis of differential metabolites in different forests. **(A)** CP forest vs. BP forest litter layers. **(B)** CP forest vs. MCBP forest litter layers. **(C)** BP forest vs. MCBP forest litter layers. **(D)** CP forest vs. BP forest soil layers. **(E)** CP forest vs. MCBP forest soil layers. **(F)** BP forest vs. MCBP forest soil layers.

Differential metabolites of litter between different forests were further analyzed (Figure 4). In the CP forests vs. BP forests, 122 differential metabolites were selected, which were 13 benzene and substituted derivatives, 44 lipids, 21 organic acids, 2 amino acids, and 26 sugars (Figure 4A). In addition, 58 metabolites were downregulated, and 64 metabolites were upregulated. In the CP forests vs. MCBP forests, 66 differential metabolites were selected, which were from 13 benzene and substituted derivatives, 19 lipids, 20 organic acids, 1 amino acid, and 12 sugars (Figure 4B). In addition, 33 metabolites were downregulated, and 33 metabolites were upregulated. In the BP forests vs. MCBP forests, 122 differential metabolites were selected, which were from 13 benzene and substituted derivatives, 45 lipids, 21 organic acids, 2 amino acids, 34 sugars, and 1 phenolic acid (Figure 4C). In addition, 58 metabolites were downregulated, and 64 metabolites were upregulated. Furthermore, the results of the random forest model showed that the top 20 metabolites were the best biomarkers in litter layers, which were cellulose and hemicellulose-degrading products such as sugars and amino acids and lignin-degrading products such as benzene and substituted derivatives (Supplementary Figure S5a).

Differential metabolites of soil between different forests were also determined (Figures 4D–F). In the CP forests vs. BP forests, 12 differential metabolites were selected, which were from 1 benzene and substituted derivative, 2 lipids, 2 polyphenols, and 6 sugars. In addition, 7 metabolites were downregulated, and 5 metabolites were upregulated (Figure 4D). In the CP forests vs. MCBP forests, 16 differential metabolites were selected, which were 1 lipid, 8 sugars, 4 amino acids, and 3 organic acids. In addition, 6 metabolites were downregulated, and 10 metabolites were upregulated (Figure 4E). In the BP forests vs. MCBP forests, 13 differential metabolites were selected, which were from 1 benzene and substituted derivative, 2 lipids, 7 sugars, and 3 amino acids. Additionally, 4 metabolites were downregulated, and 9 metabolites were upregulated (Figure 4F). The result of the random forest model indicated that the main biomarkers were derived from phenols, benzene and substituted derivatives, amino acids, and sugars, which were also from lignocellulose degradation in litter layers (Supplementary Figure S5b).

#### 3.4.2 Distribution of key metabolites

**Figure 5:**
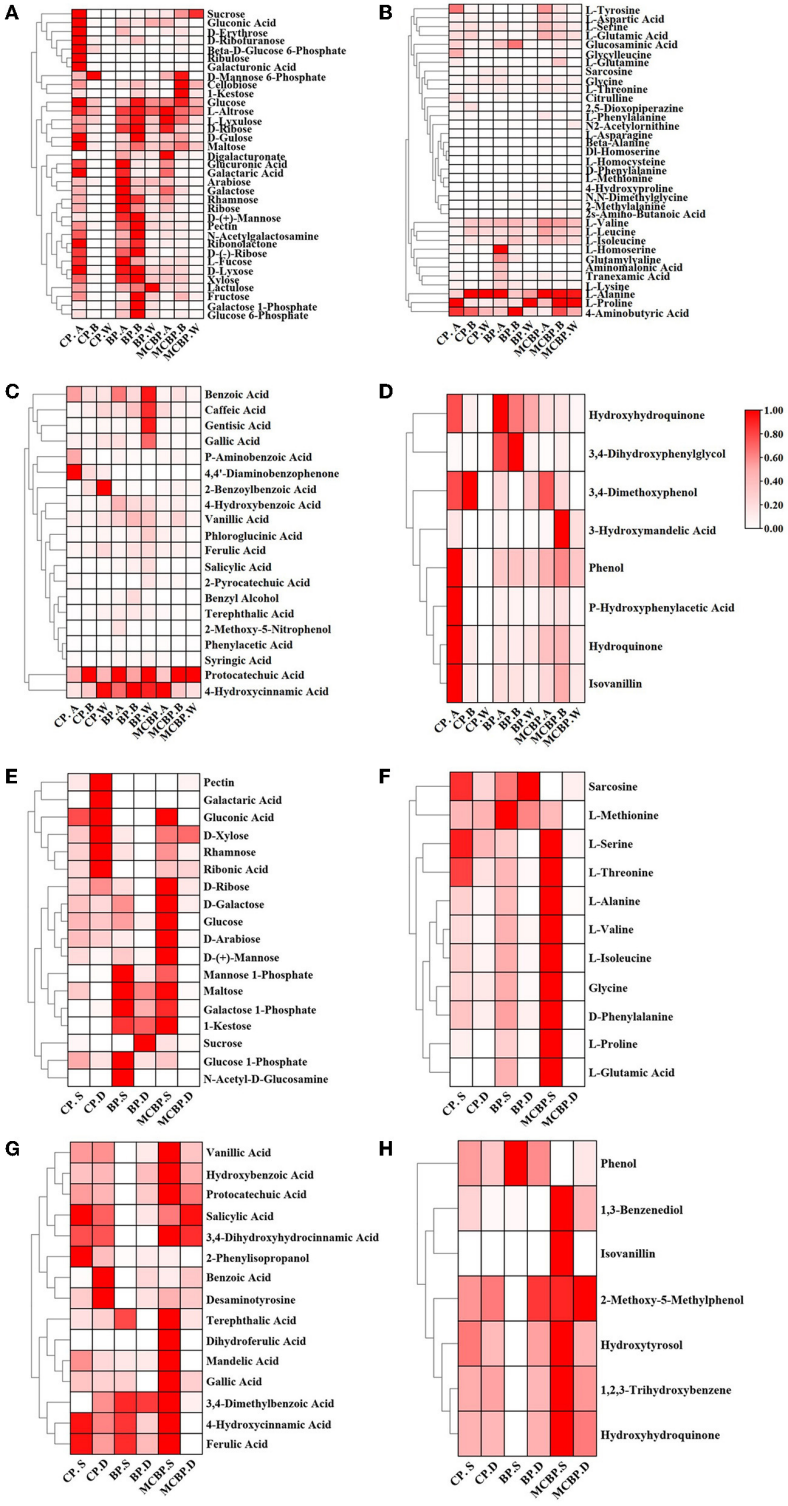
Changes in key metabolites in different forests. **(A)** Changes in carbohydrates in the litter layer. **(B)** Changes in amino acids in the litter layer. **(C)** Changes in benzene and organic acids in the litter layer. **(D)** Changes in phenols in the litter layer. **(E)** Changes in carbohydrates in the soil layer. **(F)** Changes in amino acids in the soil layer. **(G)** Changes in benzene and organic acids in the soil layer. **(H)** Changes in phenols in the soil layer.

On the one hand, the relative abundance of some sugars (including sucrose, gluconic acid, D-erythrose, galacturonic acid, and ribulose) in CP. A was higher than that in BP. A and MCBP. A, while the relative abundance of the other sugars (including D-mannose, pectin, N-acetylgalactosamine, D-ribose, D-xylose, and xylose) in BP. A and BP. B was the highest (Figure 5A). For the totally decomposed litter layers, the relative abundance of sugars was in the order of BP. W, MCBP. W, and CP. W (Figure 5A). The relative abundance of amino acids during litter decomposition showed different changes (Figure 5B). The relative abundance of L-alanine in the CP forests and MCBP forests was the highest. The relative abundance of L-proline and 4-aminobutyric acid in the BP forests and MCBP forests was higher than that in the CP forests. In addition, the relative abundance of L-tyrosine, aspartic acid, L-serine, L-glutamic acid, glycine, and L-threonine in the MCBP forests was higher than that in the CP forests and BP forests. On the other hand, phenols and benzenes are derived from the degradation of lignin. The relative abundance of benzoic acid, caffeic acid, gentisic acid, gallic acid, protocatechuic acid, and 4-hydroxycinnamic acid in the MCBP forests was the highest (Figure 5C). In addition, some benzene compounds in the BP forests were the highest, including 4-hydroxybenzoic acid, vanillic acid, phloroglucinol acid, and ferulic acid. Additionally, the relative abundance of most of the phenols in CP. A was higher than that of the BP forests and MCBP forests. The relative abundance of 3,4-imethoxyphenol and 3-hydroxymandelic acid in the undecomposed layer of MCBP forest litter was the highest (Figure 5D).

On the one hand, the relative abundance of pectin, galactaric acid, gluconic acid, xylose, rhamnose, and ribonic acid for CP. D was the highest. In addition, the relative abundance of mannose-1-phosphate, maltose, galactose-1-phosphate, 1-kestose, and glucose-1-phosphate for BP. S was the highest. The relative abundance of most sugars for MCBP. S, such as D-ribose, glucose, D-arabinose, and D-mannose, was higher than that of the BP forests and CP forests (Figure 5E). Similarly, the relative abundance of most amino acids for MCBP. S was higher than that of the BP forests and CP forests (Figure 5F). On the other hand, the relative abundance of benzene in the MCBP forests and CP forests was higher than that in the BP forests (Figure 5G). In addition, the relative abundance of hydroxyhydroquinone and isovanillin in the MCBP forest soil was the highest (Figure 5H).

### 3.5 Correlation analysis

**Figure 6:**
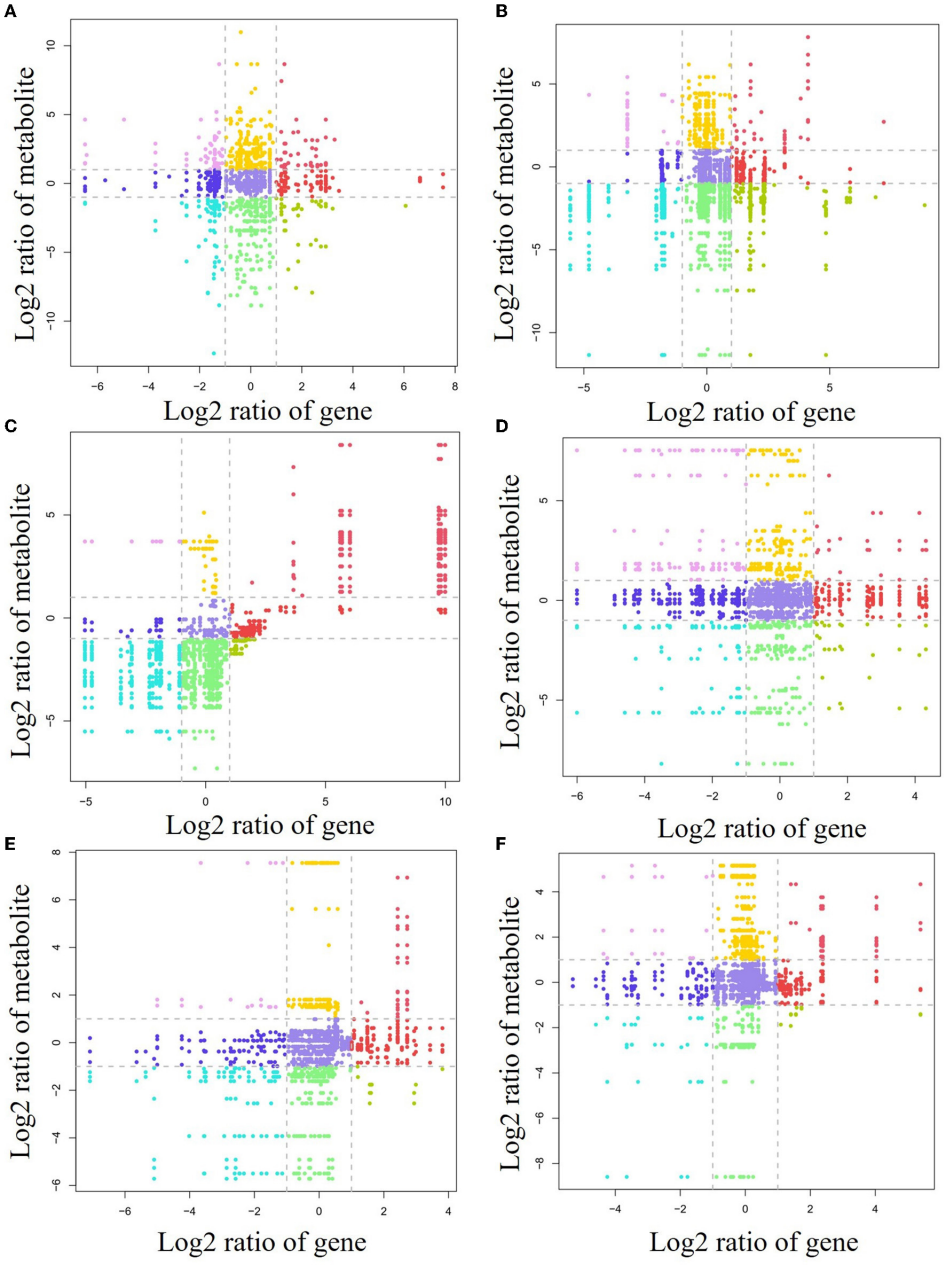
Nine-quadrant diagram of the relationship between lignocellulose-degrading enzyme encoding genes and metabolites. **(A)** CP forest vs. BP forest in the litter layer. **(B)** CP forest vs. MCBP forest in the litter layer. **(C)** BP vs. MCBP forest in the litter layer. **(D)** CP forest vs. BP forest in the soil layer. **(E)** CP forest vs. MCBP forest in the soil layer. **(F)** BP vs. MCBP forest in the soil layer.

To investigate the relationship between gene levels and metabolite levels in different forest litter and soil layers, a correlation analysis between all genes and metabolites was carried out (Figure 6). For the forest litter lignocellulosic degradation process, 21, 19, 23, and 19 genes and 49, 29, 53, and 28 related metabolites, respectively, were situated in the first quadrant, third quadrant, 7th quadrant, and 9th quadrant for the CP forests vs. BP forests (Figure 6A). For the CP forests vs. MCBP forests, 9, 14, 14, and 26 genes, and 27, 28, 96, and 102 related metabolites, respectively, were situated in the first quadrant, third quadrant, 7th quadrant, and 9th quadrant (Figure 6B). For the BP forests vs. MCBP forests, 12, 10, 20, and 26 genes, and 1, 42, 61, and 8 related metabolites, respectively, were situated in the first quadrant, third quadrant, 7th quadrant, and 9th quadrant (Figure 6C).

For forest soil layers, 45, 13, 41, and 16 genes, and 13, 11, 15, and 12 related metabolites, were situated in the first quadrant, third quadrant, 7th quadrant, and 9th quadrant in the CP forests vs. BP forests, respectively (Figure 6D). For the CP forests vs. MCBP forests, 11, 4, 42, and 8 genes, and 4, 22, 13, and 6 related metabolites, were situated in the first quadrant, third quadrant, 7th quadrant, and 9th quadrant, respectively (Figure 6E). For the BP forests vs. MCBP forests, 10, 7, 17, and 7 genes, and 6, 19, 7, and 6 related metabolites, were situated in the first quadrant, third quadrant, 7th quadrant, and 9th quadrant, respectively (Figure 6F).

### 3.6 Key factors influencing SOC formation

**Figure 7:**
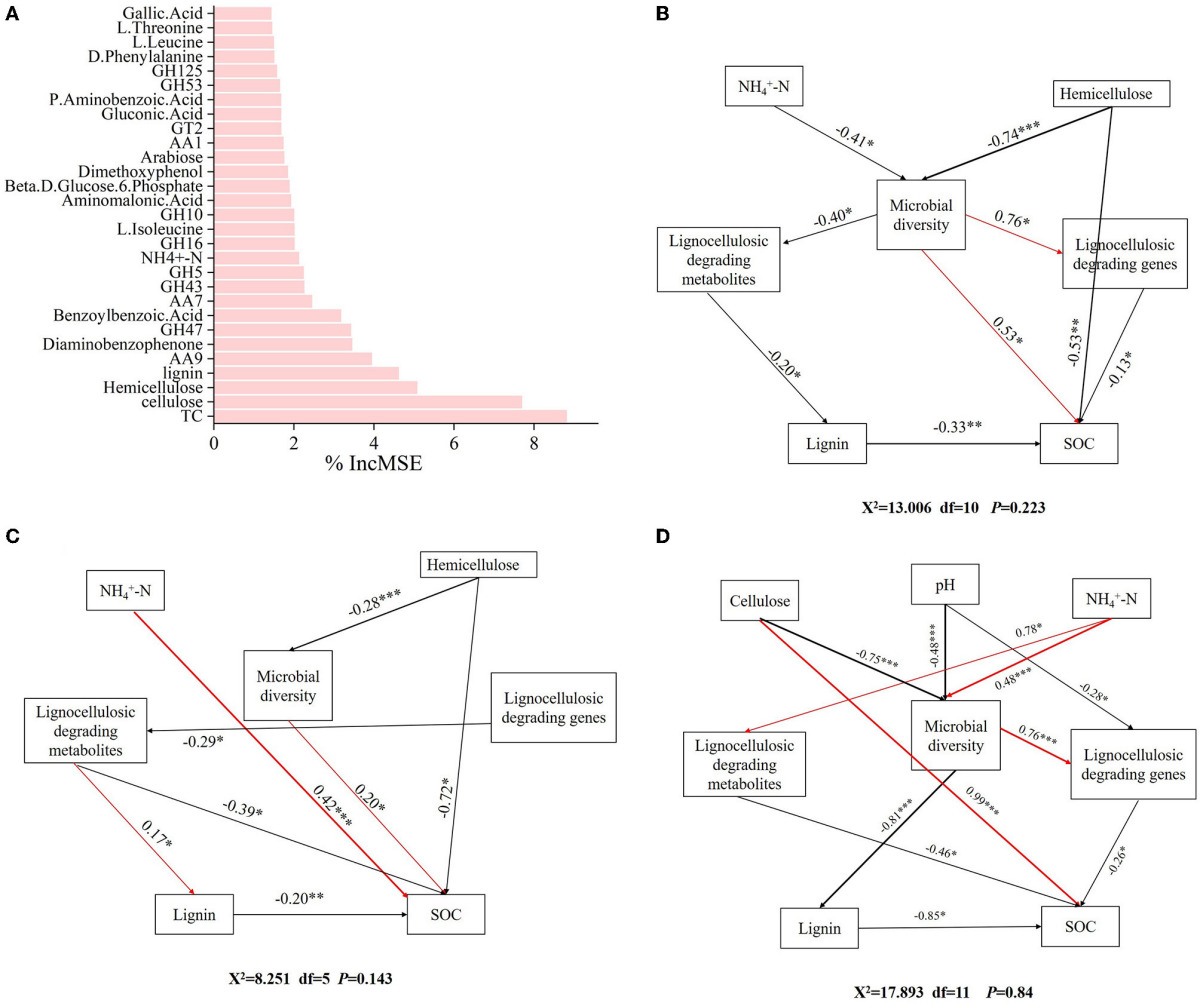
Identifying the driving factors affecting SOC formation. **(A)** Random forest analysis of the factors influencing SOC formation. Structural equation model analysis of the influencing factors of SOC formation in different forests. **(B)** CP forests. **(C)** BP forests. **(D)** MCBP forests.

The result of the random forest model explained 65.97% of the indicators in the formation of SOC. TC, cellulose, hemicellulose, lignin, *AA9, GH47*, diaminobenzophenone, benzoylbenzoic acid, *AA7, GH43*, ${\text{NH}}_{4}^{+}$-N, *GH10, GH16*, L-isoleucine, aminomalonic acid, glucose-6-phosphate, dimethoxyphenol, arabinose, *AA1*, and *GT2* were key factors influencing SOC formation (Figure 7A).

In addition, SEM was further used to analyze the microbial mechanisms of SOC formation in different forest planting patterns. For the CP forests, ammonium nitrogen (${\text{NH}}_{4}^{+}$-N) and hemicellulose had indirect effects on SOC by influencing microbial diversity (Figure 7B). In addition, microbial diversity had an indirect negative effect on SOC by influencing lignocellulose-degrading metabolites and lignin (Figure 7B). Furthermore, lignocellulose-degrading genes had a direct negative effect on SOC formation (Figure 7B). For the BP forests, ${\text{NH}}_{4}^{+}$-N and hemicellulose had a direct positive effect on SOC formation by influencing microbial diversity (Figure 7C). Lignocellulose-degrading genes had an indirect effect on SOC formation by influencing lignocellulose-degrading metabolites (Figure 7C). In addition, hemicellulose and lignin had a direct negative effect on SOC formation (Figure 7C). For the MCBP forests, cellulose and pH had direct negative effects on microbial diversity, while microbial diversity had an indirect effect on SOC formation by influencing lignocellulose-degrading genes or lignin (Figure 7D). In addition, ${\text{NH}}_{4}^{+}$-N had indirect effects on SOC formation by influencing microbial diversity and lignocellulose-degrading metabolites (Figure 7D).

## 4 Discussion

### 4.1 Lignocellulose degradation and microbial communities in different forest planting patterns during litter decomposition

The microbial diversity of litter in BP forests and MCBP forests was higher than that of CP forests, which was due to the difference in lignocellulose components, indicating that the BP litter (more cellulose and hemicellulose) was more easily decomposed than the CP litter (Prescott et al., 2000; Almeida et al., 2021). The existence of high-quality litter (readily decomposable) in the BP forest litter layers and more nitrogen and less lignin concentration in the MCBP forest litter layers resulted in rapid decomposition performance and higher microbial diversity in comparison with the CP forests, promoting nutrient turnover rates and SOC formation (Wang W. et al., 2020; Lyu et al., 2023). These higher-quality litter inputs may contain enough N to meet the microbial nutrition demands to break down high-quality litter, which has a greater priming effect than low-quality litter. The slow lignin degradation of low-quality litter (i.e., CP forests) limits microbial activities and metabolic processes (Wang W. et al., 2020).

The dominant phyla in different forests were distinctly significant (Figure 1D). This was closely associated with microbial life history strategies depending on regulated ecological functions (Jiang et al., 2021; Wu et al., 2023). Proteobacteria and Bacteroides are affiliated with r-strategy bacteria, while Actinobacteria and Acidobacteria are affiliated with k-strategy bacteria (Jiang et al., 2021). Mixed planting of forest litter increased the relative abundance of Proteobacteria, which showed a potential synergistic effect. Proteobacteria and Bacteroides preferred more degradable substrates and were sensitive to easily decomposable C sources, whereas Actinobacteria and Acidobacteria were sensitive to recalcitrant C sources (Jiang et al., 2021; Zhu et al., 2022). Proteobacteria and Bacteroides were mainly in the litter layers, while Actinobacteria and Acidobacteria were mainly in the soil layers (Figures 1C–E). In addition, the dominant fungal phylum in the CP forests was Ascomycota, while the dominant fungal phyla in the BP forests were Basidiomycota and Chytridiomycota, which was due to the difference in substrate composition, preference of litter, and strategies of microbes (Wang W. et al., 2020). The recalcitrant substrate of CP forest litter was stronger, resulting in a slower nutrient turnover rate. Ascomycota was an r-strategies, while Basidiomycota and Chytridiomycota were k-strategies, resulting in differences in lignocellulose degradation rates and SOC formation (Liu et al., 2022). Furthermore, the dominant archaeal phyla in the CP forests and BP forests were Euryarchaeota and Thaumarchaeota, respectively. In summary, the dominant bacteria of the BP forests and MCBP forests were r-strategies, while the dominant fungi of the CP forests were r-strategies, depending on microbial species metabolism strategies and unique functional microbial composition. Thus, these results supported our first hypothesis.

Cellulose and hemicellulose were more readily available to microbes, whereas lignin was difficult to degrade and was not conducive to being used by microbes (Almeida et al., 2021). In this study, the degradation of lignocellulose in BP litter and MCBP litter was more rapid than that in CP litter, indicating that the BP forest and MCBP forest litter accelerated nutrient turnover and were more conducive to SOC formation (Sheffer et al., 2015). In addition, higher priming effects in the BP forests strongly affected microbial communities and related enzymes, increased lignocellulose degradation, and decreased the SOC concentration (Lyu et al., 2023). Compared with the CP forests, the mixing of tree species accelerated the effects of litter lignocellulose degradation because higher nitrogen and lower lignin concentrations led to microbial proliferation, increased microbial diversity, and decreased priming effects, promoting SOC formation (Wang W. et al., 2020). Thus, the mixing of tree species improved the slow degradation rate of coniferous tree species and increased the functional features of coniferous tree species or broadleaved tree species, promoting SOC formation. Our results regarding the complex dynamics of lignocellulose degradation and SOC formation are just instantaneous and require long-term *in situ* observations during litter decomposition in the future.

### 4.2 Key metabolic pathways of lignocellulose degradation and SOC formation in different forest planting patterns

Lignocellulose-degrading enzymes are encoded by diverse lignocellulose-degrading genes, which act in synergistic action to degrade lignocellulose (Wang et al., 2022; Li et al., 2023). Litter substrate composition was the most critical factor affecting the variations in soil extracellular enzymes, functional genes, and microbial community composition, resulting in differences in lignocellulose-degrading products and SOC formation (Wang W. et al., 2020; Li et al., 2023). The relative abundance of cellulose-degrading genes and hemicellulose-degrading genes (GHs and PLs family genes) in BP forest litter was the highest, whereas the relative abundance of lignin-degrading genes (AAs family genes) in CP forest litter was the highest (Figure 2). The differences in the substrate composition of litter and soil properties resulted in differences in lignocellulose-degrading genes. Cellulose and hemicellulose-degrading genes of MCBP forest litter were higher than those of CP forest litter, indicating that mixed plantation forest litter exhibited a complex composition of litter, which reduced the lignin concentration of litter and increased the rate of substrate turnover, thereby increasing the relative abundance of lignocellulose-degrading genes, which was consistent with the study by Wang W. et al. (2020) and Wang Y. et al. (2020). Higher lignocellulose-degrading genes for MCBP litter could encode and secrete lignocellulose-degrading enzymes and then degrade lignocellulose (Wang W. et al., 2020). GTs family genes were used for organic C synthesis. GTs family genes in the totally decomposed litter layer and the soil layers in the BP forests and MCBP forests were the highest, indicating that the BP forests and MCBP forests increased lignocellulose degradation and promoted SOC formation. Similarly, GHs family genes (*GH1, GH2, GH6, GH39*, and *GH53*) for BP litter and some GTs family genes (*GT87, GT47, GT4, GT35, GT2*, and *GT19*) for BP soil were the highest, indicating that BP forest litter increased lignocellulose degradation and promoted SOC formation. Higher lignin concentrations in CP forest litter led to a higher abundance of AAs family genes such as *AA1, AA5, AA6, AA7*, and *AA10* (Figures 2, 3). Even though there was a higher abundance of lignin-degrading genes in CP litter, less nitrogen limited the production of ligninase, decreasing lignin degradation. In addition, lignin wrapped the surface of cellulose and hemicellulose, and the higher lignin concentration in the CP litter limited the degradation of lignocellulose, thus decreasing litter decomposition and SOC formation (Wu et al., 2023). Thus, the BP forests had stronger nutrient substrate turnover rates. The MCBP forests increased lignocellulose degradation and decreased priming effects, promoting SOC formation. The lignocellulose-degrading genes allowed the microbial communities as a whole to capture energy and nutrients from lignocellulosic biomass, which showed similar functional properties (Wang W. et al., 2020). However, a drawback of this research is that gene abundance and gene expression are not consistently correlated (Lacerda Junior et al., 2017). In the future, we need to use transcriptomics to explore the expression levels of lignocellulose-degrading genes.

Another important aspect is mainly derived from the difference in lignocellulose-degrading metabolites (Wu et al., 2023). Most of the metabolites were derived from phenols, benzene and substituted derivatives, long-chain carbon organic acids, amino acids, and sugars. These metabolites might originate from lignocellulose degradation during litter decomposition, which may be SOC precursors to promote SOC formation based on lignin-protein theory, polyphenol-protein theory, microbial synthesis theory, and carbonyl amine condensation theory (Wang et al., 2017; Wu et al., 2023). As the decomposition of litter progressed, a greater proportion of metabolites flowed to SOC, and these metabolites were microbial-derived or litter-derived (Campbell et al., 2022). The differential metabolic pathways between the CP forest and BP forest litter layers were mainly from amino acids, sugars, the TCA cycle, and the cAMP signaling pathway, indicating that variations in litter substrate composition and functional microbes resulted in differential metabolites and pathways and thereby influenced the SOC precursors and SOC formation (Wang et al., 2017; Campbell et al., 2022). In the CP forests vs. MCBP forests, differential pathways were smaller, which was due to the partly similar plant species composition with a higher degree of similarity in litter substrate composition and lower microbial diversity, resulting in the same lignocellulose-degrading products and fewer differential pathways (Campbell et al., 2022). However, the recalcitrant nature of the lignin structure in CP litter increased differential metabolites between the BP forests and MCBP forests (Figure 4C). Lignin-degrading products were derived from a variety of phenols, aldehydes, acids, etc. (Wu et al., 2023). In addition, except for the CP forest vs. BP forest and BP forest vs. MCBP forest soil layers, there were no differential pathways between the CP forest and MCBP forest soil layers, indicating that SOC formation was dominant and that the microbial community composition gradually stabilized, which in turn led to a decrease in differential metabolic pathways in the soils (Figure 4). In addition, differential pathways were mainly derived from the amino acid metabolism pathway, pentose phosphate pathway, and cAMP signaling pathway between the CP forest and BP forest soil layers and between the BP forest and MCBP forest soil layers, indicating that the differential metabolic pathway of soil was dominated by basal metabolism after lignocellulose degradation, which provided nutrients and energy to microbes and further drove the formation of SOC (Supplementary Figure S4e).

On the one hand, the differences in sugars during litter decomposition led to the differences in SOC. The relative abundance of monosaccharides (including sucrose, gluconic acid, D-erythrose, galacturonic acid, and ribulose) in CP. A was the highest, indicating that the degradation of coniferous tree species litter produced these monosaccharides (Chen et al., 2014). However, D-mannose, pectin, N-acetylgalactosamine, D-ribose, and xylose for BP. A and BP. B were the highest, indicating that the BP forests increased the microbial diversity and activities, increased the degradation of cellulose and hemicellulose, and increased the relative abundance of monosaccharides, thus promoting SOC formation (Wang W. et al., 2020). The degradation of different types of litter produced different types of sugars. The sugar produced by BP litter was mainly lignocellulose degradation, while the sugar produced by CP litter was mainly microbial metabolism (Wu et al., 2023). On the other hand, amino acids were the key metabolites in the formation of SOC during litter decomposition (Lyu et al., 2023). The relative abundance of amino acids in the BP forests and MCBP forests was higher than that in the CP forests, indicating that a higher nitrogen concentration increased the production of amino acids, which was more favorable to C metabolism (Chen et al., 2014). Additionally, L-tyrosine, aspartic acid, L-serine, L-glutamic acid, and glycine were the highest in the MCBP forest litter, indicating that mixed species forests accelerated amino acid metabolism to promote SOC formation (Chen et al., 2014; Wang W. et al., 2020). In addition, the relative abundance of L-tryptophan, L-tyrosine, and L-phenylalanine concentrations in the shikimic acid pathway was the highest for MCBP forest litter. These aromatic amino acids were basic skeletons that promote SOC formation (Wu et al., 2017). Furthermore, the phenolic acids and benzenes in MCBP forest litter were the highest, indicating that mixed-species forests accelerated lignin degradation and increased SOC precursor generation. Increasing the nitrogen concentration in the MCBP forest litter increased the production of lignin-degrading enzymes, increasing lignin degradation and producing lignin-degrading metabolites (Margida et al., 2020). More importantly, lignocellulose-degrading products of MCBP forest soil were the highest, indicating that mixed species forests promoted litter metabolite production, thus promoting SOC formation in soil layers (Wang W. et al., 2020). Even though lignocellulose-degrading genes in the BP forests were higher, metabolites in the MCBP forests were more abundant and diverse. Thus, these phenomena supported our second hypothesis that differential microbial functional genes and metabolites involved in lignocellulose degradation resulted in the difference in SOC formation in forest planting patterns.

### 4.3 Key factors influencing SOC formation in forest planting patterns

The random forest model results revealed that amino acids, lignocellulose-degrading products, and lignocellulose-degrading genes were key factors influencing SOC formation by affecting microbial activities (Zhang et al., 2018). In addition, SOC formation in forest planting patterns was influenced by different factors from two aspects: one was the direct action of microbes, and the other was the indirect production of lignocellulose-degrading metabolites by microbes or lignocellulose-degrading genes (Zhang et al., 2018). Furthermore, slight changes in environmental factors and soil properties affected the production of key microbial genes, in turn affecting lignocellulose-degrading metabolites (Chen et al., 2023). For the CP forests, ${\text{NH}}_{4}^{+}$-N, and hemicellulose were key factors affecting microbial diversity, thereby influencing SOC formation. The higher lignin concentration limited hemicellulose degradation, and the lower concentration of ${\text{NH}}_{4}^{+}$-N decreased microbial diversity and lignocellulose degradation, thus decreasing the SOC concentration (Wu et al., 2023). For the BP forests, in addition to the key factors influencing microbial diversity, lignocellulose-degrading genes had a negative correlation with lignocellulose-degrading metabolites, indicating that the BP litter increased microbial activities and the abundance of lignocellulose-degrading genes, promoting the production of lignocellulose-degrading metabolites and SOC formation (Wang W. et al., 2020). Lignocellulose-degrading genes were a key factor in promoting the production of lignocellulose-degrading metabolites in the BP forests, promoting SOC formation (Wilhelm et al., 2019). For the MCBP forests, cellulose, pH, and ${\text{NH}}_{4}^{+}$-N were key factors influencing microbial diversity, while microbial diversity had a negative correlation with lignin, thus influencing SOC formation. An increase in the nitrogen concentration of litter increased the microbial diversity and the production of functional enzymes and promoted the degradation of lignin (Keeler et al., 2009; Lyu et al., 2023). Compared to CP forest litter, MCBP forest litter increased cellulose degradation and enhanced sugar and amino acid metabolism, thus increasing lignocellulose degradation and SOC formation due to the decrease in lignin concentration of the litter (Wang W. et al., 2020). Mixed plantation forest litter increased cellulose and lignin degradation by increasing microbial activities and metabolism, thus promoting the formation and sequestration of SOC. These results supported our third hypothesis that key factors affecting the formation of SOC in different forest planting patterns were significantly different (Wang W. et al., 2020; Lyu et al., 2023).

## 5 Conclusion

BP forest litter and MCBP forest litter had higher lignocellulose degradation during litter decomposition. The SOC concentrations were higher by 38.06 and 94.43% in the BP forests and 38.55 and 20.87% in the MCBP forests for the 0–10 cm, and 10–20 cm soil layers, respectively, compared to the CP forests. Compared with the CP forests, the MCBP forests increased microbial diversity and changed microbial community structure. Additionally, gene abundances for GHs and PLs in BP forest litter were the highest, increasing the production of lignocellulose-degrading metabolites. In comparison with CP forest litter, MCBP forest litter increased the relative abundances of lignocellulose-degrading genes, accelerating the production of metabolites, proving diversified SOC formation pathways and precursors, and promoting SOC formation. More importantly, amino acid metabolism and sugar metabolism were the key factors affecting the difference in SOC concentration in different forest planting patterns. GHs and AAs family genes were key genes involved in lignocellulose degradation, while PLs family genes were key genes involved in SOC formation. The sugars produced by CP litter and BP litter were mainly involved in lignocellulose degradation metabolism and microbial metabolism, respectively. In addition, metabolites of aromatic amino acids, phenolic acids and benzene ring compounds were markers in the MCBP forests, while cellulose and hemicellulose-degrading metabolites were markers in the BP forests.

## Data availability statement

The original contributions presented in the study are included in the article/Supplementary material, further inquiries can be directed to the corresponding author. The data reported of metabonomics sequencing in this paper have been deposited in the OMIX, China National Center for Bioinformation/Beijing Institute of Genomics, Chinese Academy of Sciences (https://ngdc.cncb.ac.cn/omix: accession no.OMIX005332). The data of metagenomics sequencing presented in the study are deposited in National Center for Biotechnology Information database, and the accession number is PRJNA995878.

## Author contributions

DW: Conceptualization, Formal analysis, Funding acquisition, Software, Visualization, Writing – original draft, Writing – review & editing. CY: Conceptualization, Writing – original draft. YF: Data curation, Writing – original draft. HC: Methodology, Writing – original draft. ZL: Formal analysis, Writing – original draft. GJ: Funding acquisition, Project administration, Resources, Supervision, Writing – review & editing.

## References

[B1] Almeida L. Souza I. Hurtarte L. Teixeira P. Inagaki T. Silva I.. (2021). Forest litter constraints on the pathways controlling soil organic matter formation. Soil. Biol. Biochem. 163, 108447. 10.1016/j.soilbio.2021.108447

[B2] Campbell T. Ulrich D. Toyoda J. Thompson J. Munsky B. Albright M.. (2022). Microbial communities influence soil dissolved organic carbon concentration by altering metabolite composition. Front. Microbiol. 12, 799014. 10.3389/fmicb.2021.799014 35126334 PMC8811196

[B3] Cao T. Fang Y. Chen Y. Kong X. Yang J. Alharbi H.. (2022). Synergy of saprotrophs with mycorrhiza for litter decomposition and hotspot formation depends on nutrient availability in the rhizosphere. Geoderma. 410, 115662. 10.1016/j.geoderma.2021.115662

[B4] Cassart B. Basia A. Titeux H. Andivia E. Ponette Q. (2017). Contrasting patterns of carbon sequestration between *Gilbertiodendron dewevrei* monodominant forests and *Scorodophloeus zenkeri* mixed forests in the Central Congo basin. Plant Soil. 414, 309–326. 10.1007/s11104-016-3130-8

[B5] Chen H. Gurmesa G. Liu L. Zhang T. Fu S. Liu Z.. (2014). Effects of litter manipulation on litter decomposition in a successional gradients of tropical forests in southern China. PLoS ONE. 9, e99018. 10.1371/journal.pone.0099018 24901698 PMC4047082

[B6] Chen J. Xiao Q. Xu D. Li Z. Chao L. Li X.. (2023). Soil microbial community composition and co-occurrence network responses to mild and severe disturbances in volcanic areas. Sci. Total Environ. 901, 165889. 10.1016/j.scitotenv.2023.165889 37524180

[B7] Detain J. R'emond C. Rodrigues C. Harakat D. Besaury L. (2022). Co-elicitation of lignocelluloytic enzymatic activities and metabolites production in an Aspergillus-Streptomyces co-culture during lignocellulose fractionation. Curr. Res. Microb. Sci. 3, 100108. 10.1016/j.crmicr.2022.100108 35243445 PMC8861581

[B8] Ding K. Zhang Y. Wang L. Ge S. Zhang Y. Yang Q.. (2023). Forest conversion from pure to mixed *Cunninghamia lanceolata* plantations enhances soil multifunctionality, stochastic processes, and stability of bacterial networks in subtropical southern China. Plant Soil. 488, 411–429. 10.1007/s11104-023-05983-y

[B9] Dunn W. B. Broadhurst D. Begley P. Zelena E. Francis-McIntyre S. Anderson N.. (2011). Procedures for large-scale metabolic profiling of serum and plasma using gas chromatography and liquid chromatography coupled to mass spectrometry. Nat. Protoc. 6, 1060–1083. 10.1038/nprot.2011.335 21720319

[B10] Hu T. Wang X. Zhen L. Gu J. Zhang K. Wang Q.. (2019). Effects of inoculating with lignocellulose-degrading consortium on cellulosedegrading genes and fungal community during co-composting of spent mushroom substrate with swine manure. Bioresour. Technol. 291, 121876. 10.1016/j.biortech.2019.121876 31377509

[B11] Irene F. T. Felipe B. Teresa H. Petra B. Hans H.. (2014). The role of lignin and cellulose in the carbon-cycling of degraded soils under semiarid climate and their relation to microbial biomass. Soil Biol. Biochem. 75, 152–160. 10.1016/j.soilbio.2014.04.007

[B12] Jiang S. Xing Y. Liu G. Hu C. Wang X. Yan G.. (2021). Changes in soil bacterial and fungal community composition and functional groups during the succession of boreal forests. *Soil* Biol. Biochem. 161, 108393. 10.1016/j.soilbio.2021.108393

[B13] Jones C. McConnell C. Coleman K. Cox P. Falloon P. Jekinson D.. (2005). Global climate change and soil carbon stocks; predictions from two contrasting models for the turnover of organic carbon in soil. Global Change Biol. 11, 154–166. 10.1111/j.1365-2486.2004.00885.x

[B14] Keeler B. Hobbie S. Kellogg L. (2009). Effects of long-term nitrogen addition on microbial enzyme activity in eight forested and grassland sites: implications for litter and soil organic matter decomposition. Ecosystems.12, 1–15. 10.1007/s10021-008-9199-z

[B15] Lacerda Junior G. Noronha M. Sousa S. Cabral L. Domingos D. Saber M.. (2017). Potential of semiarid soil from Caatinga biome as a novel source for mining lignocellulose-degrading enzymes. FEMS Micro. Biol. Ecol. 93, fiw248. 10.1093/femsec/fiw248 27986827

[B16] Li J. Niu X. Wang P. Yang J. Liu J. Wu D.. (2023). Soil degradation regulates the effects of litter decomposition on soil microbial nutrient limitation: evidence from soil enzymatic activity and stoichiometry. Front. Plant Sci. 13, 1090954. 10.3389/fpls.2022.1090954 36684742 PMC9853160

[B17] Li R. Zhu L. Wang Y. Zhu Y. (2022). Metagenomic insights into environmental risk of field microplastics in an urban river. Water Res. 223, 119018. 10.1016/j.watres.2022.119018 36057234

[B18] Liang C. Amelung W. Lehmann J. Kästner M. (2019). Quantitative assessment of microbial necromass contribution to soil organic matter. Glob. Chang. Biol. 25, 3578–3590. 10.1111/gcb.14781 31365780

[B19] Liang C. Schimel J. P. Jastrow J. D. (2017). The importance of anabolism in microbial control over soil carbon storage. Nat. Microbiol. 2, 17105. 10.1038/nmicrobiol.2017.105 28741607

[B20] Liu T. Wu X. Li H. Ning C. Li Y. Zhang X.. (2022). Soil quality and r–K fungal communities in plantations after conversion from subtropical forest. Catena. 219, 106584. 10.1016/j.catena.2022.106584

[B21] Lyu M. Homyak P. Xie J. Peñuelas J. Ryan M. Xiong X.. (2023). Litter quality controls tradeoffs in soil carbon decomposition and replenishment in a subtropical forest. J. Ecol. 111, 2181–2193. 10.1111/1365-2745.14167

[B22] Margida M. Lashermes G. Moorhead D. (2020). Estimating relative cellulolytic and ligninolytic enzyme activities as functions of lignin and cellulose content in decomposing plant litter. *Soil* Biol. Biochem. 141, 107689. 10.1016/j.soilbio.2019.107689

[B23] Martyna M. Xavier G. David S. Corinne R. Yves R. Philippe D.. (2017). Optimization of a metatranscriptomic approach to study the lignocellulolytic potential of the higher termite gut microbiome. BMC Genomis. 18, 681. 10.1186/s12864-017-4076-9 28863779 PMC5580439

[B24] Nemergut D. Cleveland C. Wieder W. Washenberger C. Townsend A. (2010). Plot-scale manipulations of organic matter inputs to soils correlate with shifts in microbial community composition in a lowland tropical rain forest. *Soil* Biol. Biochem. 42, 2153–2160. 10.1016/j.soilbio.2010.08.011

[B25] Nie Y. Wang M. Zhang W. Ni Z. Hashidoko Y. Shen W. (2018). Ammonium nitrogen content is a dominant predictor of bacterial community composition in an acidic forest soil with exogenous nitrogen enrichment. Sci. Total Environ. 624, 407–415. 10.1016/j.scitotenv.2017.12.142 29262382

[B26] Prescott C. E. Zabek L. M. Staley C. L.. (2000). Decomposition of broadleaf and needle litter in forests of British Columbia: influences of litter type, forest type, and litter mixtures. Can. J. Forest Res. 30, 1742–1750. 10.1139/x00-097

[B27] Roeland S. Moretti M. Amorim J. Branquinho C. Fares S. Morelli F.. (2019). Towards an integrative approach to evaluate the environmental ecosystem services provided by urban forest. J. For. Res. 30, 1981–1996. 10.1007/s11676-019-00916-x

[B28] Sato Y. Kumagai T. Kume A. Otsuki K. Ogawa S. (2004). Experimental analysis of moisture dynamics of litter layers-the effects of rainfall conditions and leaf shapes. Hydrol. Process. 18, 3007–3018. 10.1002/hyp.5746

[B29] Sheffer E. Canham C. Kigel J. Perevolotsky A. (2015). Countervailing effects on pine and oak leaf litter decomposition in human-altered Mediterranean ecosystems. Oecologia 177, 1039–1051. 10.1007/s00442-015-3228-3 25680333

[B30] Shi S. Herman D. He Z. Pett-Ridge J. Wu L. Zhou J.. (2018). Plant roots alter microbial functional genes supporting root litter decomposition. *Soil* Biol. Biochem. 127, 90–99. 10.1016/j.soilbio.2018.09.013

[B31] Thomas B. Quentin S. Thibaut L. Aurore R. (2020). Single and mixed feedstocks biorefining: comparison of primary metabolites recovery and lignin recombination during an alkaline process. Front. Chem. 8, 479. 10.3389/fchem.2020.00479 32582644 PMC7292014

[B32] Valášková V. Šnajdr J. Bittner B. Cajthaml T. Merhautova V. Hofrichter M.. (2007). Production of lignocellulose-degrading enzymes and degradation of leaf litter by saprotrophic basidiomycetes isolated from a Quercus petraea forest. *Soil* Biol. Biochem. 39, 2651–2660. 10.1016/j.soilbio.2007.05.023

[B33] Veres Z. Kotrocz,ó Z. Fekete I. Tóth J. Lajtha K. Townsend K.. (2015). Soil extracellular enzyme activities are sensitive indicators of detrital inputs and carbon availability. Appl. Soil Ecol. 92, 18–23. 10.1016/j.apsoil.2015.03.006

[B34] Voeller K. Bílek H. Kreft J. Dostalkova A. Kozliak E. Kubatova A. (2017). Thermal carbon analysis enabling comprehensive characterization of lignin and its degradation products. Sustain. Chem. Eng. 5, 10334–10341. 10.1021/acssuschemeng.7b02392

[B35] Wang K. Gao P. Geng L. Liu C. Zhang J. Shu C. (2022). Lignocellulose degradation in *Protaetia brevitarsis* larvae digestive tract: refining on a tightly designed microbial fermentation production line. Microbiome. 10, 90. 10.1186/s40168-022-01291-2 35698170 PMC9195238

[B36] Wang M. Cui J. Liu H. Xu X. (2023). Characterization of soil microbial biomass carbon and nitrogen in four forest types of shushan urban forest park. Forests. 14, 1498. 10.3390/f14071498

[B37] Wang T. Tian Z. Bengtson P. Tunlid A. Persson P. (2017). Mineral surface-reactive metabolites secreted during fungal decomposition contribute to the formation of soil organic matter. Environ. Microbiol. 19, 5117–5129. 10.1111/1462-2920.13990 29124857

[B38] Wang W. Chen D. Sun X. Zhang Q. Koide R. Insam H.. (2019). Impacts of mixed litter on the structure and functional pathway of microbial community in litter decomposition. Appl. Soil Ecol. 144, 72–82. 10.1016/j.apsoil.2019.07.006

[B39] Wang W. Zhang Q. Sun X. Chen D. Insam H. Koide R.. (2020). Effects of mixed-species litter on bacterial and fungal lignocellulose degradation functions during litter decomposition. *Soil* Biol. Biochem. 141, 107690. 10.1016/j.soilbio.2019.107690

[B40] Wang Y. Li T. Li C. Song F. (2020). Differences in microbial community and metabolites in litter layer of plantation and original korean pine forests in North Temperate Zone. Microorganisms. 8, 2023. 10.3390/microorganisms8122023 33348766 PMC7765820

[B41] Wilhelm R. Singh R. Eltis L. Mohn W. (2019). Bacterial contributions to delignification and lignocellulose degradation in forest soils with metagenomic and quantitative stable isotope probing. ISME J. 13, 413–429. 10.1038/s41396-018-0279-6 30258172 PMC6331573

[B42] Wu D. Qu F. Li D. Zhao Y. Li X. Niu S.. (2022). Effect of Fenton pretreatment and bacterial inoculation on cellulose-degrading genes and fungal communities during rice straw composting. Sci. Total Environ. 806, 151376. 10.1016/j.scitotenv.2021.151376 34740666

[B43] Wu D. Ren H. Zhao Y. Wei Z. Li J. Song C. (2023). Effect of Fenton-like reactions on the hydrolysis efficiency of lignocellulose during rice straw composting based on genomics and metabolomics sequencing. J. Clean. Prod. 396, 136493. 10.1016/j.jclepro.2023.136493

[B44] Wu J. Zhao Y. Qi H. Zhao X. Yang T. Du Y.. (2017). Identifying the key factors that affect the formation of humic substance during different materials composting. Bioresource Technol. 244, 1193–1196. 10.1016/j.biortech.2017.08.100 28863988

[B45] Xie Q. Liu B. Dong W. Li J. Wang D. Liu Z.. (2023). Comparative transcriptomic and metabolomic analyses provide insights into the responses to NaCl and Cd stress in *Tamarix hispida*. Sci. Total Environ. 884, 163889. 10.1016/j.scitotenv.2023.163889 37142042

[B46] Xu M. Zhi R. Jian J. Feng Y. Han X. Zhang W. (2022). Changes in soil organic C fractions and C pool stability are mediated by C-degrading enzymes in litter decomposition of *Robinia pseudoacacia* plantations. Microb. Ecol. 86, 1189–1199. 10.1007/s00248-022-02113-6 36123554

[B47] Xu X. Sun Z. Hao Z. Bian Q. Wei K. Wang C. (2021). Effects of urban forest types and traits on soil organic carbon stock in Beijing. Forests. 12, 394. 10.3390/f12040394

[B48] Zhang X. Li X. Ji X. Zhang Z. Zhang H. Zha T.. (2021). Elevation and total nitrogen are the critical factors that control the spatial distribution of soil organic carbon content in the shrubland on the Bashang Plateau, China. Catena. 204, 105415. 10.1016/j.catena.2021.105415

[B49] Zhang Z. Wang J. Li B. (2018). Determining the influence factors of soil organic carbon stock in opencast coal-mine dumps based on complex network theory. Catena. 173, 433–444. 10.1016/j.catena.2018.10.030

[B50] Zhou Y. Clark M. Su J. Xiao C. (2015). Litter decomposition and soil microbial community composition in three Korean pine (Pinus koraiensis) forests along an altitudinal gradient. Plant Soil. 386, 171–183. 10.1007/s11104-014-2254-y

[B51] Zhu K. Wang Q. Zhang Y. Zarif N. Ma S. Xu L. (2022). Variation in soil bacterial and fungal community composition at different successional stages of a Broad-Leaved Korean pine forest in the Lesser Hinggan Mountains. Forests. 13, 625. 10.3390/f13040625

