# Supplementary material for: Effect of forest planting patterns on the formation of soil organic carbon during litter lignocellulose degradation from a microbial perspective
Source: Front Microbiol. 2023 Dec 22;14:1327481. doi: 10.3389/fmicb.2023.1327481 (PMC10771852; doi:10.3389/fmicb.2023.1327481)
Supplement: Supplementary file 1 [file Data_Sheet_1.docx]

**Effect of forest planting patterns on the formation of soil organic carbon during litter lignocellulose degradation from a microbial perspective**

Di Wu^1,2,3^, Changwei Yin^1^, Yuxin Fan^1^, Haiyu Chi^1^, Zhili Liu^1,2,3^, Guangze Jin^1,2,3*^

^1^ Center for Ecological Research, Northeast Forestry University, Harbin 150040, China

^2^ Key Laboratory of Sustainable Forest Ecosystem Management-Ministry of Education, Northeast Forestry University, Harbin 150040, China

^3^ Northeast Asia Biodiversity Research Center, Northeast Forestry University, Harbin 150040, China

* Correspondence author: Guangze Jin

Northeast Forestry University, Harbin 150040, China

E-mail address: [taxus@126.com](mailto:taxus@126.com).

Supporting Information Includes:

1 table

5 figures

4 texts

Table S1 The significant differences in genes related to C metabolism in different forests

|  | Downregulated | FC | P | Upregulated | FC | P |
| --- | --- | --- | --- | --- | --- | --- |
| CP forest vs BP forest | GH7 | -1.70 | 0.00001 | GT101 | 2.72 | 0.0001 |
|  | AA4 | -0.83 | 0.0008 | GT48 | 3.64 | 0.0004 |
|  | AA12 | -1.64 | 0.009 | GH43 | 0.43 | 0.033 |
|  | GH27 | -0.56 | 0.024 | GT77 | 1.75 | 0.05 |
|  | GT12 | -1.64 | 0.04 |  |  |  |
|  | AA2 | -1.84 | 0.042 |  |  |  |
| CP forest vs. MCBP forest | GT17 | -1.16 | 0.007 | GT48 | 3.42 | 0.0029 |
|  | GT12 | -2.14 | 0.02 | GT39 | 0.80 | 0.015 |
|  | GH8 | -0.74 | 0.023 | AA10 | 0.77 | 0.011 |
|  | AA14 | -1.83 | 0.04 | GH53 | 0.97 | 0.021 |
|  |  |  |  | GT7 | 0.85 | 0.03 |
|  |  |  |  | GT22 | 0.522 | 0.038 |
| BP forest vs. MCBP forest | GT101 | -3.01 | 0.0002 | AA10 | 0.89 | 0.003 |
|  | AA14 | -1.96 | 0.027 | GT22 | 0.65 | 0.009 |
|  |  |  |  | GH47 | 0.94 | 0.010 |
|  |  |  |  | GT7 | 0.96 | 0.014 |
|  |  |  |  | AA4 | 0.58 | 0.037 |
|  |  |  |  | GT105 | 1.65 | 0.050 |

Note: FC: Fold Change


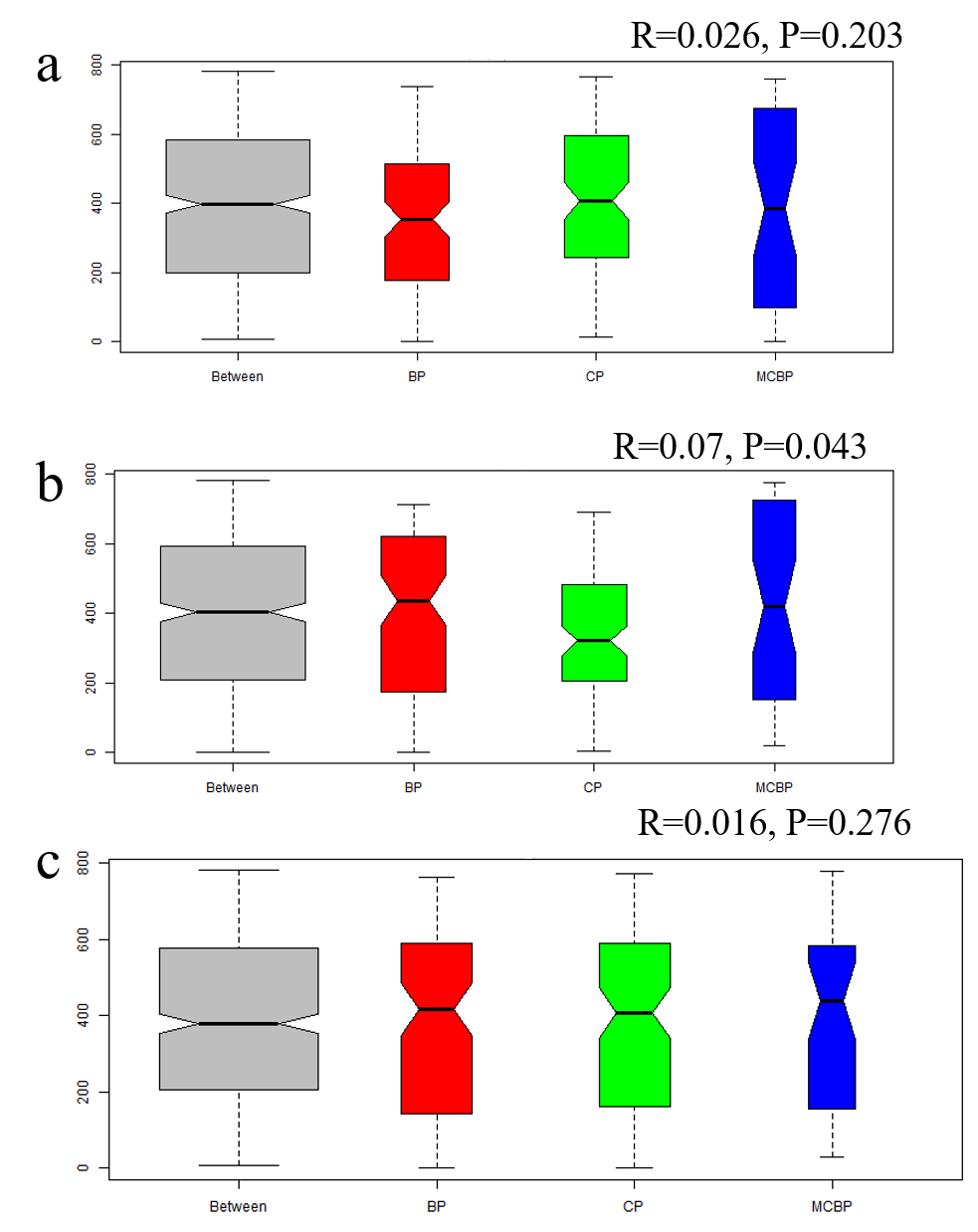


**Figure S1** Statistical differences of microbial communities among different forests. (a) archaeal communities (b) bacterial communities (c) fungal communities.


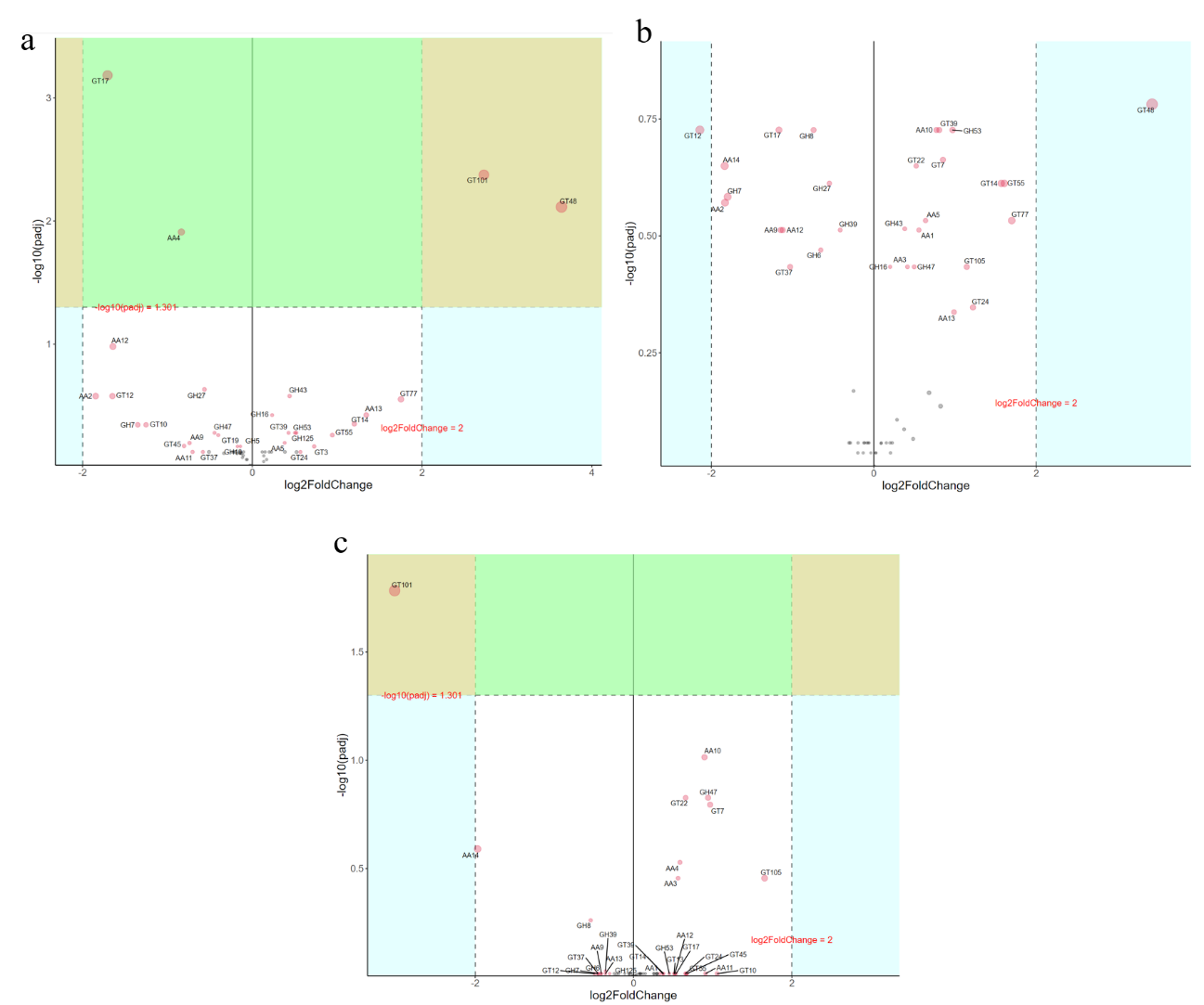


**Figure S2** The differences genes related to C metabolism in different forests. (a) CP forest vs. BP forest (b) CP forest vs. MCBP forest (c) BP forest vs. MCBP forest.


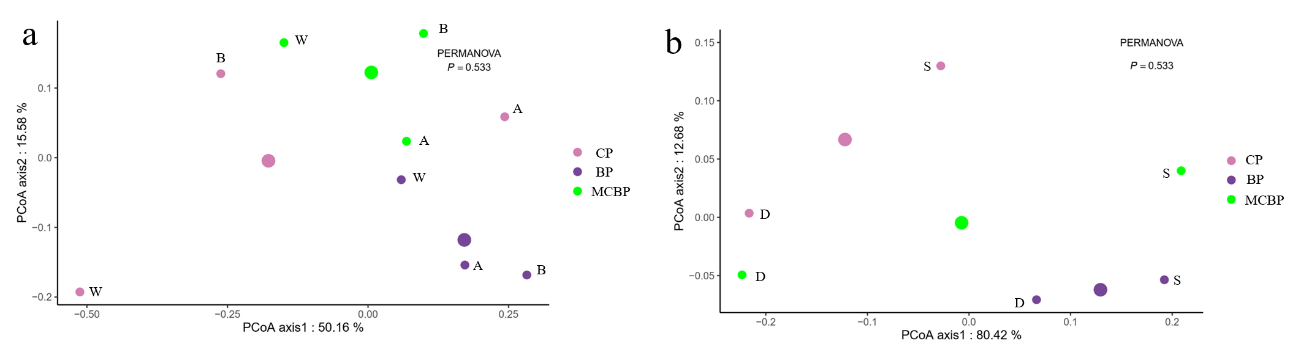


**Figure S3** The significant difference analysis of the litter decomposition metabolites and soil C synthesis metabolites based on Bray-Curtis distances.


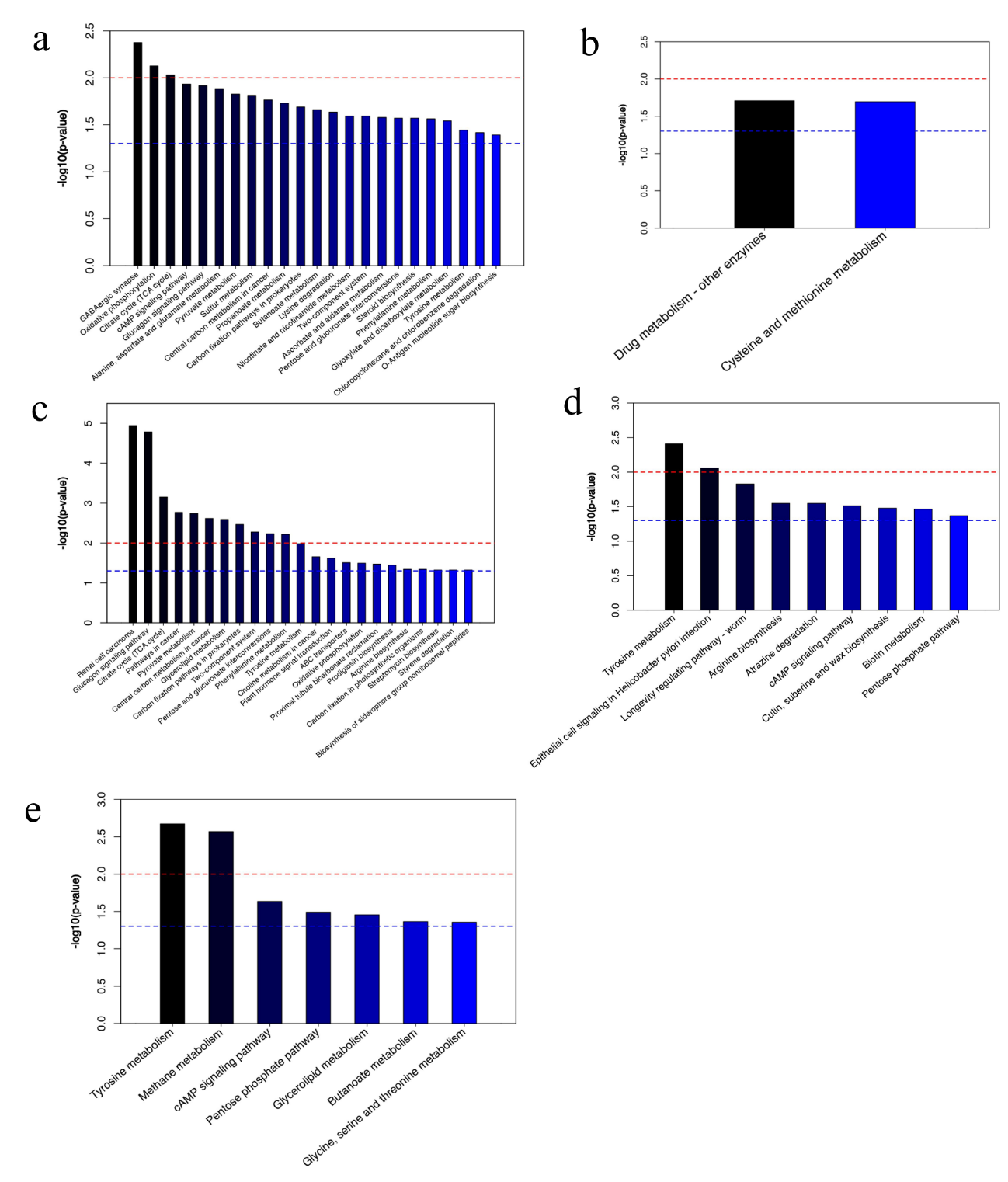


**Figure S4** Differential metabolic pathways were enriched among different forest based on *P*<0.05. (a) CP forest vs. BP forest litter layers (b) CP forest vs. MCBP forest litter layers (c) BP forest vs. MCBP forest litter layers (d) CP forest vs. BP forest soil layers (e) BP forest vs. MCBP forest soil layers.


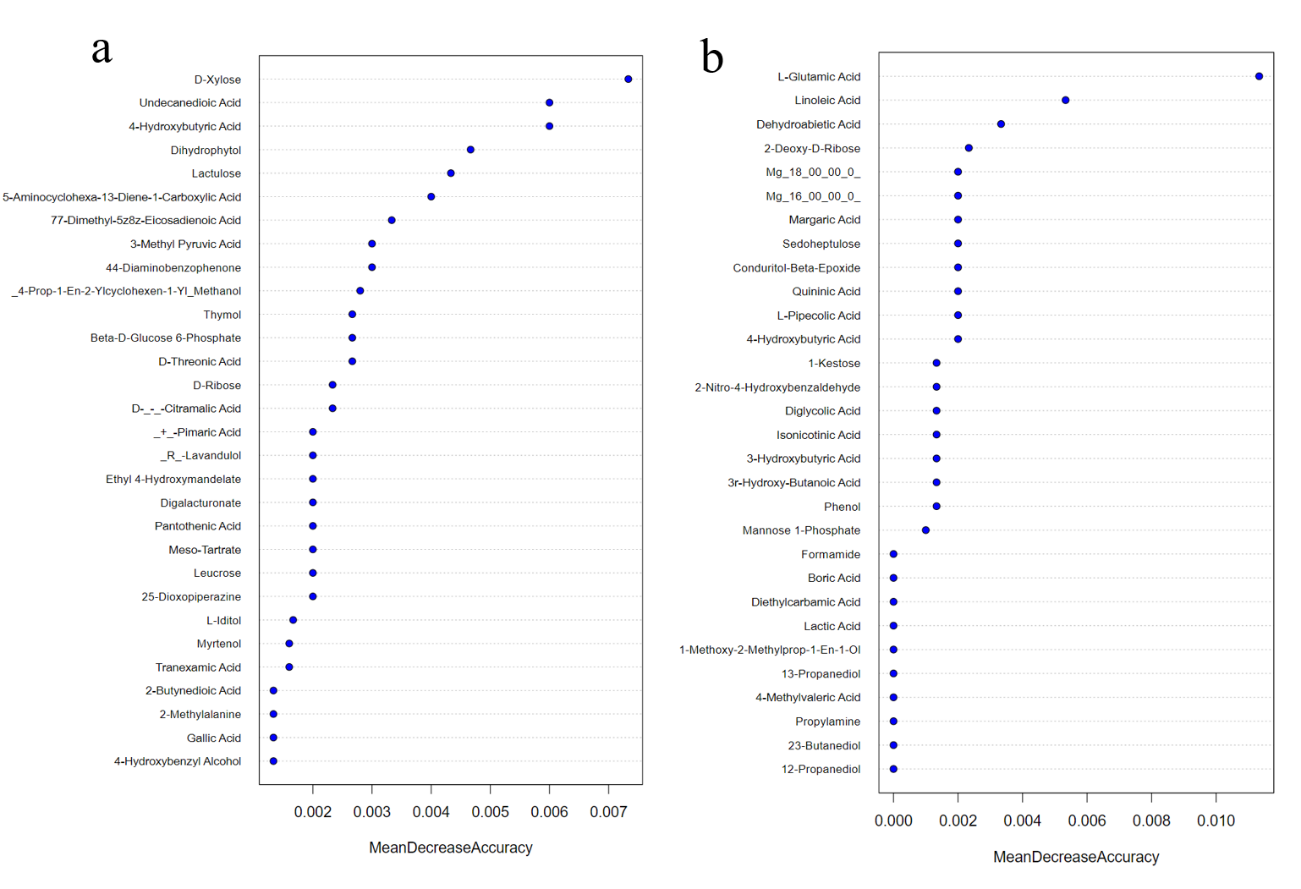


**Figure S5** Random Forest model analysis of best biomarkers in all forest (a) litter layers and (b) soil layers.

Text: 3.2.2

The significant differences in genes related to C metabolism in different forests were further analyzed (Table S1, Figure S2). In the CP forest vs. BP forest, the GT17, AA4, AA12, GH27, GT12, AA2 genes were downregulated, while the GT101, GT48, GH43, GT77 genes were upregulated (Figure S2a). In the CP forest vs. MCBP forest, the GT17, GT12, GH8, AA14 genes were downregulated, while GT48, GT39, AA10, GH53, GT7, GT22 genes were upregulated (Figure S2b). In the BP forest vs. MCBP forest, the GT101, AA14 genes were downregulated, while AA10, GT22, GH47, GT7, AA4, GT105 genes were upregulated (Figure S2c).

Text: 3.4.1

Differential metabolites of litter between different forests were further analyzed. 23 differential pathways were enriched between CP forest and BP forest, including central carbon metabolism in cancer, propanoate metabolism, lysine degradation, amino acids, sugars, TCA cycle and cAMP signaling pathway (Fig. S4a). 2 differential pathways were enriched, such as drug metabolism, cysteine and methionine metabolism between CP forest and MCBP forest (Fig. S4b). In addition, 23 differential pathways were enriched between BP forest and MCBP forest (such as the TCA cycle, pyruvate metabolism, phenylalanine metabolism, tyrosine metabolism, etc.) (Fig. S4c).

In addition, differential metabolites of soil between different forests were also determined. Differential enrichment pathways were further analyzed (Fig. S4d). Tyrosine metabolism, epithellial cell signaling in helicobacter pylori infection, longevity regulating pathway, arginine biosynthesis, atrazine degradation, cAMP signaling pathway, cutin, suberine and wax biosynthesis, biotin metabolism, pentose phosphate pathway were differential pathways between CP forest and BP forest. The differential pathways between CP forest and MCBP forest were not observed. In addition, tyrosine metabolism, methane metabolism, cAMP signaling pathway, pentose phosphate pathway, glycerolipid metabolism, butanoate metabolism, glycine, serine, and threonine metabolism were differential pathways between BP forest and MCBP forest (Fig. S4e).

Analysis of physical and chemical indicators

The pH value in all forests showed an increasing at first and then decreasing trend during litter decomposition (Table 1). Mixed planting forest increased pH value in litter and soil layers. The NH_4_^+^-N and NO_3_^-^-N concentrations showed a decreasing trend during litter decomposition. Similarly, mixed planting forest increased NH_4_^+^-N and NO_3_^-^-N concentrations in litter and soil layers (Table 1).

Text 4.2

The nine-quadrant plot revealed that the number of lignocellulose-degrading genes related to corresponding metabolites for CP forest vs. BP forest was the highest, which was due to significant differences of litter substrate composition and production of functional genes (P < 0.05) (Xie et al., 2022). In addition, the relatively easier decomposition of litter for BP forest increased the similarity of lignocellulose degrading products for MCBP forest, leading to more similar lignocellulose-degrading genes and associated metabolites (Wang et al., 2020a). Species-rich litter for MCBP forest increased sugar metabolism and amino acid metabolism, changed the quality of litter inputs, and then changed the formation of SOC (Campbell et al., 2022).

1. Xie, L., Yin, C., 2022. Seasonal variations of soil fungal diversity and communities in subalpine coniferous and broadleaved forests. Sci Total Environ. 846, 157409.
2. Campbell, T., Ulrich, D., Toyoda, J., Thompson, J., Munsky, B., Albright, M., Bailey, V., Tfaily, M., Dunbar, J., 2022. Microbial communities influence soil dissolved organic carbon concentration by altering metabolite composition. Front Microbiol. 12, 799014.
3. Wang, W., Zhang, Q., Sun, X., Chen, D., Insam, H., Koide, R., Zhang, S., 2020a. Effects of mixed-species litter on bacterial and fungal lignocellulose degradation functions during litter decomposition. Soil Biol Biochem. 141, 107690.
